# Sex-specific regulation of angiogenin in Alzheimer’s disease

**DOI:** 10.1038/s41380-026-03656-7

**Published:** 2026-06-02

**Authors:** Marko Jörg, Lukas Walz, Sebastian Nathal, Marco Kristen, Christine Lietz, Max Müller, Vu Thu Thuy Nguyen, Nicolas Ruffini, Marie-Luise Winz, Susanne Gerber, Kristina Endres, Mark Helm, Kristina Friedland

**Affiliations:** 1 Institute of Pharmaceutical and Biomedical Sciences, Johannes Gutenberg University, Mainz, Germany; 2 Department of Psychiatry and Psychotherapy, University Medical Center of the Johannes Gutenberg University, Mainz, Germany; 3 Institute of Human Genetics, University Medical Center Johannes Gutenberg University, Mainz, Germany; 4 Campus Zweibrücken, University of Applied Sciences Kaiserslautern, Zweibrücken, Germany

**Keywords:** Neuroscience, Psychiatric disorders

## Abstract

Alzheimer’s disease (AD) is a heterogeneous neurodegenerative disorder, highlighting the need to identify novel molecular regulators for effective treatment development. Angiogenin (ANG), a stress-responsive ribonuclease that inhibits apoptosis by generating 5′-tRNA fragments, is a candidate whose expression and regulation in AD is not understood. Here, we investigated ANG expression and regulation using AD cell and animal models, postmortem human brain tissue, and transcriptomic datasets (*n* = 645). We found that ANG is dysregulated in AD in a sex-dependent manner, altering downstream levels of 5′-tiRNA^Gly-GCC^. Our analysis revealed female-specific molecular subtypes, absent in males: Subtype 1 featured low ANG levels with increased inflammation and neuronal death; subtype 2 exhibited higher ANG expression and intermediate pathology; subtype 3, marked by the highest ANG levels, showed reduced inflammation, slower cognitive decline, and extended survival. These findings position ANG as a key modulator of neuroinflammation and apoptosis in AD, highlighting its potential as a treatment strategy.

**Figure:**
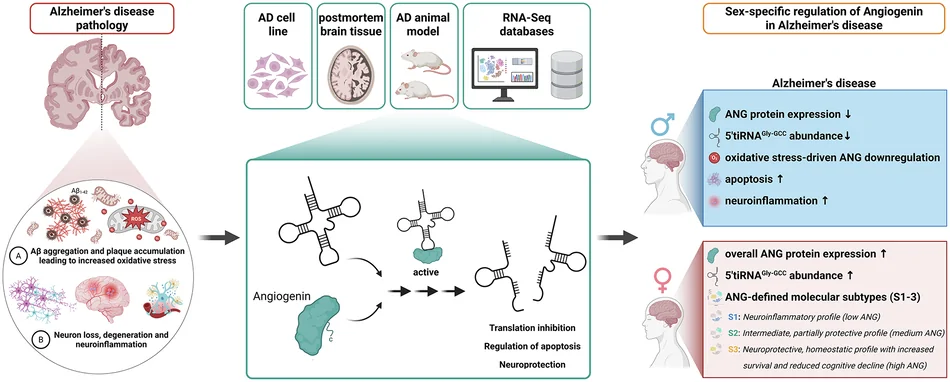

## Introduction

Alzheimer’s disease (AD) is a progressive neurodegenerative disorder characterized by synaptic and neuronal loss, cognitive decline, and the accumulation of amyloid-β (Aβ) plaques and tau pathology [1–4]. Dysregulated oxidative stress, mitochondrial dysfunction, and chronic neuroinflammation play central roles in AD pathogenesis, ultimately creating a vicious cycle of metabolic impairment and neurodegeneration [5–10]. Increasing evidence indicates that sex-specific differences in disease manifestation and rates of cognitive decline are key contributors to AD heterogeneity [11–13]. Females have a higher lifetime risk of AD and experience more rapid cognitive decline and greater neuropathological burden than males, including higher levels of pathology at autopsy even among cognitively normal individuals [14–17]. These pronounced sex differences point to underlying biological variation in stress-response and inflammatory pathways; however, the molecular mechanisms driving these disparities still remain unresolved.

Given these observations, increasing attention has focused on identifying still-unknown upstream molecular regulators that may modulate disease heterogeneity in AD. One such candidate is angiogenin (ANG), a 14.1 kDa ribonuclease that is broadly expressed across human tissues [18–20]. Beyond its originally described pro-angiogenic function, ANG regulates ribosomal RNA transcription, and cellular stress adaptation [19–31]. Under oxidative stress, ANG cleaves mature tRNAs to generate 30–40 nucleotide tRNA-derived fragments (tiRNAs), which modulate translation and promote stress-granule formation. Among these, 5′-tiRNA^Gly-GCC^ is particularly abundant and has been shown to inhibit apoptosis by interfering with apoptosome formation [20–25, 31]. Stress-induced ANG activity can therefore markedly increase tiRNA levels and regulate cell fate decisions [32–38]. ANG has also been linked to neuroprotection, and mutations that reduce its RNase activity or structural stability have been associated with neurodegenerative diseases, including amyotrophic lateral sclerosis (ALS), Parkinson’s disease (PD), and potentially AD [19, 20, 26–30, 36, 39, 40]. However, the role of ANG in AD remains unresolved, partly because its small molecular mass (14.1 kDa) and basic isoelectric point (pI 9.5) limit its detection in proteomics workflows, while low transcript abundance and sequencing depth constraints reduce its representation in large-scale transcriptomic datasets.

Because ANG is tightly coupled to cellular stress responses, emerging evidence links it to two major AD-relevant pathways: the inflammasome-mediated neuroinflammation and oxidative defense pathway. Activation of the NOD-like receptor family pyrin domain-containing 3 (NLRP3) inflammasome in microglia, which can be triggered by Aβ aggregates, promotes the release of pro-inflammatory cytokines such as interleukin-1β (IL-1β), contributing to neuroinflammatory injury in AD [41–43]. ANG-derived tiRNAs have been shown to suppress NLRP3 activation, directly modulating inflammatory signaling [43]. Inhibition of NLRP3 signaling enhances Aβ clearance and preserves cognitive function by promoting a phagocytic, metabolically active microglial phenotype [41, 42]. In parallel, ANG supports neuronal survival and antioxidant defense in part through activation of the transcription factor nuclear factor erythroid 2-related factor 2 (Nrf2) [36, 39, 40]. Nrf2 regulates antioxidant capacity by inducing target genes, including heme oxygenase-1 (HO-1), NAD(P)H:quinone oxidoreductase-1 (NQO1), and catalase (CAT), while Keap1 acts as a negative regulator of Nrf2 signaling [44–47]. Beyond its antioxidant functions, Nrf2 also modulates inflammatory responses, further linking oxidative stress and neuroinflammation in AD [47–50].

Together, these observations raise three key questions, which we addressed using various cell and animal models of AD, postmortem human brain samples, and large-scale transcriptomic datasets: (1) Is the expression of ANG and the abundance of tRNA fragments altered in a sex-specific manner during AD pathogenesis? (2) What are the molecular mechanisms regulating ANG in this context? (3) How is ANG regulation associated with downstream inflammatory and apoptotic pathways in AD? For the first time, our data reveal sex-dependent dysregulation of ANG protein expression in AD, accompanied by altered levels of 5′-tiRNA^Gly-GCC^ and distinct inflammatory and oxidative-stress profiles in females. Notably, these changes were not observed in males, suggesting sex-specific patterns of ANG regulation and disease progression.

## Materials and methods

### Study population

Postmortem cortical brain tissue samples (*n* = 26) were obtained from the Netherlands Brain Bank (NBB, Amsterdam). In addition, transcriptomic data from previously published studies were analyzed (*n* = 619). All procedures were conducted in accordance with the ethical guidelines and approval of the respective institutions. Detailed information about data access is provided in the respective Methods section.

### Human postmortem cortical brain samples

All human postmortem brain samples were obtained from the Netherlands Brain Bank (NBB, Amsterdam, The Netherlands) under project number 1341. Informed consent for brain donation and the use of tissue and clinical data for research was obtained from all donors or their next of kin, in accordance with NBB ethical guidelines. A total of 26 brain samples were analyzed, comprising 8 female and 6 male AD cases, and 6 female and 6 male non-demented controls (Table 1). The age of individuals ranged from 71 to 102 years. Detailed information is provided in Supplementary Information (Supplementary Table S2).

**Table 1:** Demographic and neuropathological characteristics of NBB postmortem brain samples.

	Non-demented controls	Confirmed AD patients
Number of samples	12	14
Male/Female	6/6	6/8
Age [years] (Mean ± SD)		
males	88.6 ± 10.1	82.5 ± 7.4
females	81.5 ± 6.3	84.5 ± 7.5
Braak Stage (Mean ± SD)	2.5 ± 1.0	4.7 ± 0.6

### Human databases

Data from the Aging, Dementia, and Traumatic Brain Injury (ADTBI) Study (primary publication: Miller J.A. et al.) were obtained from http://aging.brain-map.org/download/index [51]. In addition, RNA-seq data from the ROSMAP study were accessed via Synapse (accession: syn23446022) under approved data use conditions. Further details on data access and analysis are provided in the Supplementary Information.

### Cell culture

Human embryonic kidney (HEK293) cells and stably overexpressing APPwt HEK293 cells were cultured as previously described [52, 53]. Additional methodological details are provided in the Supplementary Information.

### Aβ_1-42_ oligomerization

HEK293 control cells were treated with 100 nM oligomeric Aβ1–42 for 24 h, with oligomerization confirmed by Thioflavin T staining [54]. For more details, please refer to the Supplementary Information.

### Preparation of protein extracts

For Western blot analysis, cells were washed, lysed in RIPA buffer, and protein concentration was determined by the Bradford assay, with further details provided in the Supplementary Information.

### RNA Extraction from cultured cells and postmortem brain tissue

Total RNA was extracted from cultured cells and postmortem brain tissue using TRI Reagent^TM^, with additional details provided in the Supplementary Information.

### Synthesis of tRNA fragment hybridization probes, tiRNA mimic and inhibitor

A synthetic DNA hybridization probe targeting the 5’-tiRNA^Gly-GCC^, tiRNA^Gly-GCC^ mimic and tiRNA^Gly-GCC^ inhibitor was designed based on tRNA reference sequences from gtRNAdb and synthesized by biomers.net GmbH (Ulm, Germany) [55]. The probe as well as tRNA mimic and inhibitor sequences, are provided in the Supplementary Information (Supplementary Table S1).

### Western blot analysis

Proteins (20–40 µg per lane) were separated by SDS-PAGE, transferred to PVDF membranes, and processed as described by Heiser et al. [56], with detailed information on reagents, procedure and antibodies provided in the Supplementary Information.

### Real-Time quantitative PCR (RT-qPCR)

RT-qPCR was performed according to the manufacturer’s protocol. Additional methodological details are provided in the Supplementary Materials.

### Radioactive [^32^P]-labeled Northern blotting

For each sample, 10 µg of total RNA were separated by denaturing PAGE, transferred to membranes, immobilized, and prepared for hybridization, with detailed protocols for procedures, probe labeling, detection, and data analysis provided in the Supplementary Information.

### Caspase-Glo® 3/7 assay

Caspase activity was measured according to the manufacturer’s protocol. Additional methodological details are provided in the Supplementary Information.

### Transcriptomic analysis

Gene expression data were obtained from the ROSMAP study via Synapse.org (https://www.synapse.org/Synapse:syn23446022) under controlled access. Detailed information on data analysis is provided in the Supplementary Information.

### mRNA sequencing of postmortem brain tissue

Total RNA from NBB postmortem brain tissue was extracted as previously described. RNA library preparation and bulk mRNA sequencing were outsourced to Novogene (Novogene GmbH, Germany).

### GO pathway analysis

Detailed protocols for Gene Ontology (GO) pathway analysis are provided in the Supplementary Information.

### Survival rates and hazard ratios

Kaplan-Meier survival curves with 95% confidence bands were generated and compared using log-rank tests, while hazard ratios were estimated by Cox proportional-hazards modeling; further methodological details are provided in the Supplementary Information.

### Statistical analysis

All data were analyzed using standard statistical procedures in GraphPad Prism v10.4.2 (GraphPad Software, La Jolla, CA, USA). More information is provided in Supplementary Information.

### Figures and graphs

All illustrations were created using BioRender.com (premium plan including publication license). All graphs were created using GraphPad Prism 10.4.2. (GraphPad Software, La Jolla, CA, USA).

## Results

### Sex-specific alterations in ANG mRNA, protein, and tRNA-derived fragment expression in AD

**Fig. 1:**
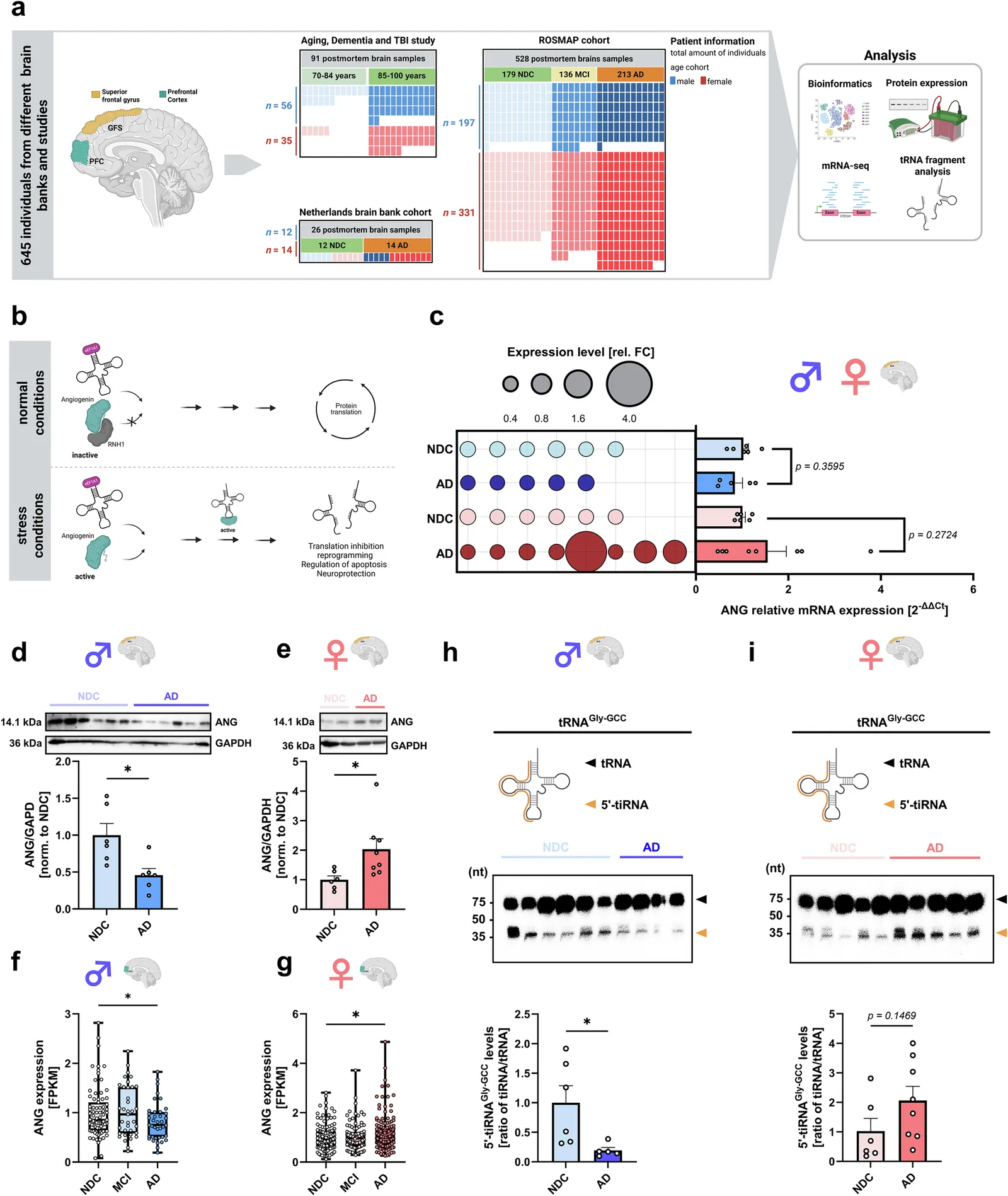
Sex-specific Alzheimer’s disease-associated changes in ANG expression and 5′-tiRNA half levels in the human prefrontal cortex. **a** Overview of cohort composition and analytical workflow. 645 donors across three postmortem brain studies composition of human (*Netherlands Brain Bank, postmortem brain tissue; Aging, Dementia and TBI Study, transcriptomic study; ROSMAP cohort, transcriptomic study*) provided prefrontal cortex (PFC; superior frontal gyrus and dorsolateral prefrontal cortex) tissue from male and female individuals. Postmortem brain tissue as well as study metadata (age, sex, clinical diagnosis) were collected, and samples were subjected to (i) bioinformatic analysis, (ii) protein expression analysis using Western Blot, (iii) quantitative PCR and RNA-seq for mRNA profiling. NBB postmortem brain tissue was also used for tRNA half profiling. **b** Model of ANG-mediated tRNA cleavage. Under basal (“normal”) conditions, ANG remains inactive, bound to its inhibitor RNH1, with no further binding to tRNAs. Under stress conditions, ANG becomes active, cleaves mature tRNAs into 5′- and 3′-tRNA halves, thereby inhibiting canonical translation and promoting stress-adaptive responses (e.g., translation reprogramming, apoptosis regulation, neuroprotection). **c** Relative ANG mRNA expression in NDC vs. AD donors stratified by sex. ANG levels are expressed as relative fold change using the ΔΔCT method (gray scale dot size indicates fold change; blue: male; red: female). bars represent mean ± SEM, *n* = 5–8 per group. No significant change was detected across groups. **d, e** ANG protein levels in NDC and AD PFC for (**d**) males and (**e**) females, assessed by Western blot analysis. In males, ANG levels are significantly decreased in AD patients, whereas in females, ANG levels are significantly increased compared to their respective NDC controls. Representative blots and quantification of ANG/GAPDH ratios (normalized to NDC) are shown; *n* = 6–8 per group; Student’s unpaired t-test, (*p < 0.05). **f,** **g** Analysis of ANG mRNA transcript levels (FPKM) in NDC and AD patients for (**f**) males and (**g**) females using mRNA-seq data from the ADTBI and ROSMAP cohorts. ANG levels are significantly downregulated in AD male patients (f), while being upregulated in AD female patients (**g**); *n* = 39–144 per group, ordinary one-way ANOVA, followed by Tukey’s multiple comparisons test (*p < 0.05). **h,** **i** [^32^P]-Northern blot analysis of tRNA^Gly-GCC^ cleavage in NDC vs. AD PFC from (**h**) males and (**i**) females, as in (**d**, **e**). Consistent with ANG expression patterns, 5′-tiRNAGly-GCC half levels were significantly decreased in males and showed a tendency to increase in females. Representative autoradiographs and quantification of 5′-tRNA half/full-length tRNA ratio (mean ± SEM); *n* = 5–8 per group, Student’s unpaired t-test, (*p < 0.05). Uncropped images of Western blot membranes and [^32^P]-labeled Northern blots are provided in the Supplementary Information.

ANG is known to play a protective role in cellular stress responses by promoting ribonucleolytic cleavage of tRNAs into small fragments such as 5′-tiRNA^Gly-GCC^, which help to suppress global translation, limit oxidative damage, and support cell survival under stress conditions. Even though ANG functions are well-characterized, it has not been systematically studied in the context of AD. Therefore, we first asked whether ANG expression and the downstream levels of 5′-tiRNA^Gly-GCC^ are altered in a sex-specific manner in AD. To address this, we performed RT-qPCR, Western blotting, and [^32^P]-Northern blotting on postmortem cortical brain tissue from the Netherlands Brain Bank (NBB), including samples from non-demented elderly individuals (non-demented controls, NDC) and patients diagnosed with mild cognitive impairment (MCI) or AD, as well as brain tissue from an AD mouse model. To validate our findings, we also analyzed large-scale RNA-seq data from the Aging, Dementia, and TBI (ADTBI) study and the Rush Orders Study and the Memory and Aging Project (ROSMAP) cohort, altogether comprising 645 individuals (Fig. 1a) [51, 57, 58]. The ADTBI study provides comprehensive neuropathological, molecular, and transcriptomic characterization within an aged and AD population-based cohort, while ROSMAP is a longitudinal clinical-pathological study focused on aging and neurodegenerative diseases [51, 57, 58]. The basic mechanism of ANG involves the cleavage of mature tRNAs to generate functional 5′-tiRNAs. These tRNA halves suppress apoptosis by inhibiting cytochrome c-mediated apoptosome formation, while also contributing to global translation inhibition and neuroprotection under stress (Fig. 1b).

We first asked whether ANG expression is altered during AD progression. ANG mRNA levels were assessed in postmortem cortical tissue from NDC and AD patients, stratified by sex using RT-qPCR. While no statistically significant differences were detected, a trend toward decreased ANG mRNA in males and increased expression in females was observed, with the female cohort displaying a notable range including individuals with extremely high and distinctly lower ANG levels (Fig. 1c). Western blot analysis of the same samples showed a significant reduction in ANG protein in male AD patients and a significant increase in female AD patients compared to their respective NDCs indicating a distinct, potentially sex-specific expression pattern (Figs. 1d, e; S1a shows the evaluation of antibody specificity). To further explore this observation, ANG protein levels from male and female AD patients were also analyzed on the same blot to allow direct visualization of sex-related differences independent of NDC comparison, revealing consistently lower ANG levels in male AD cases relative to female AD cases (Fig. S1b). Given the limited sample size of our AD cohort, we further validated these findings by analyzing large-scale RNA-seq data from the ADTBI and ROSMAP datasets, applying the same sex-stratified approach. ANG mRNA levels were significantly lower in male AD patients and significantly higher in female AD patients relative to respective NDCs (Fig. 1f, g), confirming a sex-specific dysregulation of ANG in AD. In addition, ANG mRNA expression data from male and female AD patients in the ROSMAP and ADTBI cohorts were plotted directly against each other, without normalization to NDC samples, to visualize sex-dependent differences at the transcript level (Fig. S1c, d). To further explore this phenomenon, we used the 5×FAD mouse model of familial AD, which overexpresses human APP and PSEN1 with five AD mutations under the neuron-specific Thy1 promoter [59, 60]. These mice develop Aβ plaques by the age of 2–4 months and exhibit neuroinflammation and neurodegeneration by 5–6 months [61, 62]. We assessed ANG protein levels in cortical tissue from 3- and 12-month-old 5×FAD mice and wild-type controls. While no differences in ANG levels were observed at 3 months (Supplementary Fig. S2a), at 12 months, ANG levels were significantly reduced in males and increased in females compared with wild-type controls, mirroring the distinct sex-specific pattern observed in human samples (Supplementary Fig. S2b). To assess tissue specificity, we analyzed ANG protein levels in peripheral organs (liver, kidney, lung, spleen) of wild-type and 5×FAD animals. No significant alterations were observed in young or aged transgenic animals in the respective organs, compared to their wild-type control (Supplementary Fig. S2c–f), suggesting that ANG dysregulation might be specific to the central nervous system. Given ANG’s known ribonuclease activity toward tRNAs, we next evaluated 5′-tiRNA^Gly-GCC^ levels in postmortem brain samples as a proxy for ANG activity, using [^32^P]-Northern blotting. The tRNA^Gly-GCC^ is a prominent ANG cleavage target and a well-characterized stress-induced tiRNA. In male AD patients, 5′-tiRNA^Gly-GCC^ levels were significantly reduced compared to NDCs (Fig. 1h), while females showed an upward trend (Fig. 1i). These results align with the observed ANG protein changes and support the existence of a sex-specific regulatory pattern in the ANG-tiRNA axis during AD progression.

### Oxidative stress suppresses ANG expression and reduces its anti-apoptotic function

Having demonstrated ANG dysregulation in the AD cortex, we investigated its underlying regulatory mechanisms using AD-related cell models. We first assessed ANG expression in response to oxidative stress induced by complex I dysfunction, followed by analysis under direct Aβ exposure. To model cellular oxidative stress, an important hallmark of AD, we treated human embryonic kidney (HEK293) cells with rotenone, a mitochondrial complex I inhibitor that induces mitochondrial dysfunction by impairing oxidative phosphorylation [63–65].

**Fig. 2:**
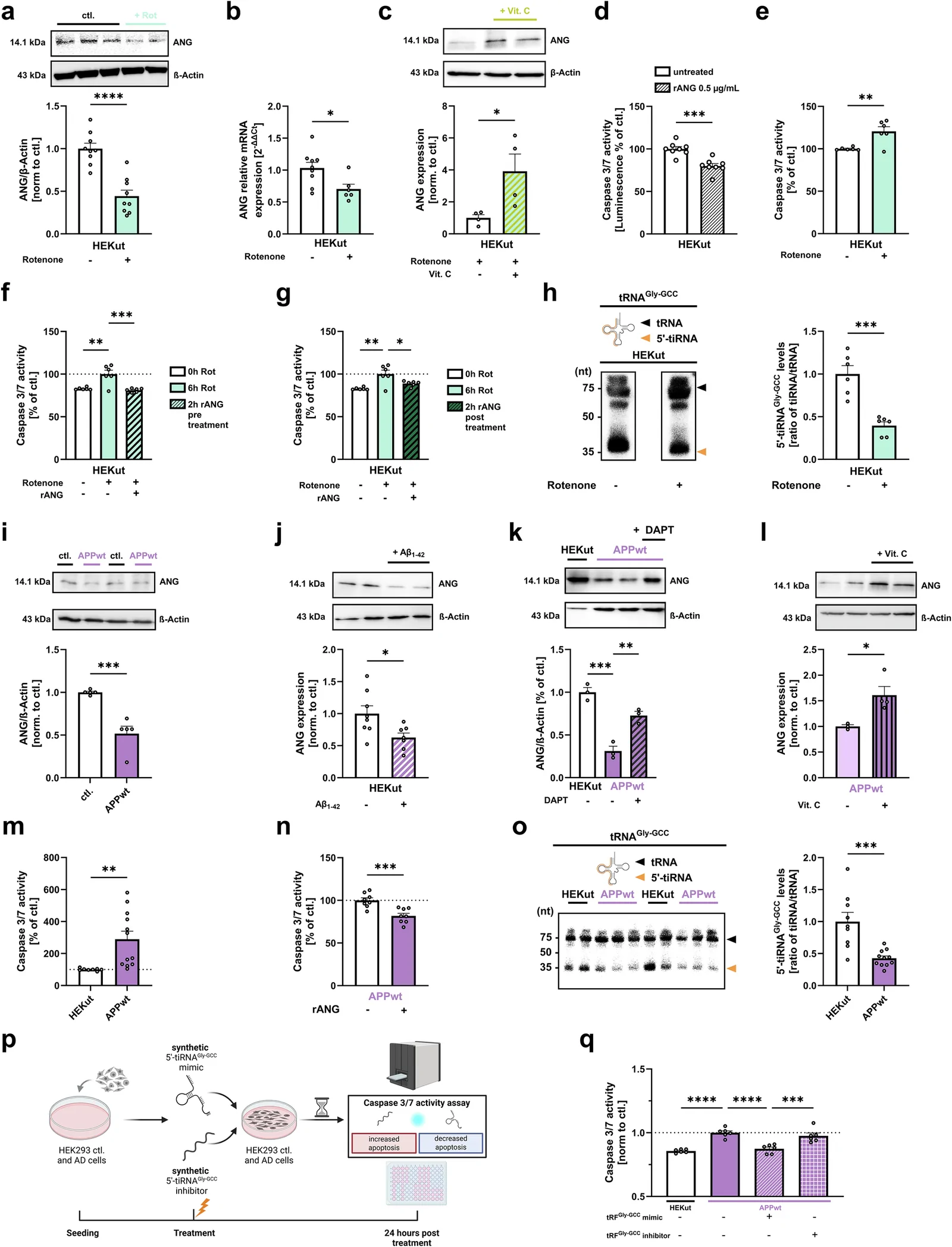
ANG protein expression regulation, and tRNA cleavage in response to cellular stress in an Alzheimer’s Disease cell model. **a** Western blot analysis of ANG protein levels in HEK293 control cells following treatment with 5 µM of mitochondrial complex I inhibitor rotenone, for 6 h, compared to solvent-treated controls. β-actin serves as a loading control. Rotenone-treated HEK293 cells revealed significantly reduced ANG protein levels compared to solvent control. *n* = 9 per group, Student’s unpaired t-test, (****p < 0.0001). **b** Quantification of ANG mRNA expression from RT-qPCR analysis, normalized to solvent-treated control, reveals a significant decrease in ANG levels upon rotenone treatment. ß-Actin was used as a reference gene. *n* = 6–9 per group, Student’s unpaired t-test, (*p < 0.05). **c** ANG protein expression measured by Western blot showing upregulation of ANG upon treatment with 1 mM of the antioxidant Vitamin C. *n* = 4 per group, Stude*n*t’s unpaired t-test, (*p < 0.05). **d** Basal caspase 3/7 activity assay in HEK293 control cells demonstrates decreased apoptotic signaling following rANG supplementation. *n* = 8 per group, Student’s unpaired t-test, (**p < 0.001). **e** Quantification of cas*p*ase 3/7 activity rev**e**aled increased apoptotic signaling following rotenone exposure. *n* = 6 per group, Student’s unpaired t-test, (**p < 0.01). **f** Caspase 3/7 activity assay in HEK293 control cells demonstrates that pretreatment with human rANG can protect cells from rotenone-induced complex I dysfunction. *n* = 6 per group, Student’s unpaired t-test, (**p < 0.01, ***p < 0.001). **g** Post-treatment with human rANG can recover cells from rotenone-induced complex I dysfunction even after rotenone damage, leading to reduced caspase 3/7 activity. *n* = 6 per group, Student’s unpaired t-test, (*p < 0.05, **p < 0.01). **h** [^32^P]-Northern blot analysis of tRNA^Gly-GCC^ in HEK293 control cells treated with rotenone, showing the reduced appearance of the respective 5′-tiRNA half alongside full-length tRNA. *n* = 6 per group, Student’s unpaired t-test, (***p < 0.001). **i** Western blot analysis of ANG protein expression revealed significant decrease in protein levels in HEK APPwt AD cell model compared to HEK293 control cells. *n* = 5 per group, Student’s unpaired t-test, (***p < 0.001). **j** Western Blot analysis revealed significantly reduced ANG protein levels in HEK293 control cells treated with oligomeric Aβ_1-42_. β-actin was used as loading control. *n* = 7–8 per group, Student’s unpaired t-test, (*p < 0.05). **k** Treatment with 1 µM of the γ-secretase inhibitor DAPT in HEK APPwt cells rescued ANG expression, increasing it to 70% of expression in HEK293 control cells. β-actin was used as a loading control. *n* = 3 per group, Student’s unpaired t-test, (**p < 0.01, ***p < 0.001). **l** ANG protein expression measured by Western Blot showing significant upregulation at the ANG level upon treatment with 1 mM of the antioxidant Vitamin C in HEK APPwt cells. *n* = 3–4 per group, Student’s unpaired t-test, (*p < 0.05). **m** Basal caspase 3/7 activity in the HEK AD cell model (APPwt) compared to HEK293 control cells revealed significantly increased activity of caspase 3/7, indicating increased rates of apoptosis. *n* = 8–12 per group, Student’s unpaired t-test, (**p < 0.01). **n** Treatment with human rANG in HEK APPwt cells significantly reduces caspase 3/7 activity. *n* = 8 per group, Student’s unpaired t-test, (**p < 0.001). **o** [^32^P]-Northern blot analysis of 5’tiRNA^GlyGCC^ revealed significantly reduced levels in HEK APPwt cells compared to control cells. *n* = 9–11 per group, Student’s unpaired t-test, (***p < 0.001). **p** Schematic representation of synthetic tRNA fragment mimic and inhibitor experiments using caspase 3/7 activity as rate for apoptosis. A mimic for the 5’tiRNA^GlyGCC^ as well as an inhibitor, was designed to imitate or inhibit its physiological cellular function. **q** Caspase 3/7 activity in HEK293 control and HEK APPwt cells transfected with synthetic 5’tiRNA^GlyGCC^ mimic or inhibitor. Treatment with the synthetic mimic demonstrates the impact on apoptotic pathways, showing reduced caspase 3/7 activity in HEK APPwt cells. *n* = 6 per group, ordinary one-way ANOVA, (**p < 0.001, ***p < 0.0001). Uncropped images of Western blot membranes and [^32^P]-labeled Northern blots are provided in the Supplementary Information.

To further investigate ANG regulation, we first examined its protein expression across varying rotenone incubation times (Supplementary Fig. S3). Western Blot and RT-qPCR analysis of rotenone-treated HEK293 cells revealed reduced ANG mRNA and protein levels, indicating that mitochondrial stress suppresses ANG expression at both transcriptional and translational levels (Fig. 2a, b). Co-treatment with vitamin C restored ANG protein levels, thereby implicating ROS as key mediators of ANG downregulation (Fig. 2c). We then investigated the functional consequences of ANG dysregulation, focusing on its role in regulating apoptosis under cellular stress. Using caspase-3/7 activity as a marker of apoptotic signaling, we found that treatment with human recombinant ANG (rANG) significantly reduced caspase activity in HEK293 cells, supporting its anti-apoptotic function (Fig. 2d). Conversely, rotenone treatment, which induces mitochondrial dysfunction, increased caspase activity, consistent with enhanced apoptosis (Fig. 2e). To determine if ANG could protect against rotenone-induced apoptosis, we measured caspase-3/7 activity following different ANG treatment conditions. We found that both pre-treatment with rANG prior to rotenone exposure and post-treatment with rANG after rotenone exposure significantly attenuated the rotenone-induced increase in caspase activity (Fig. 2f, g). These findings indicate that ANG not only prevents but may also rescue cells from oxidative stress-induced apoptosis. To determine whether the levels of 5′-tiRNA^Gly-GCC^ are affected by mitochondrial dysfunction, we assessed its abundance by [^32^P]-Northern blotting. As expected, 5′-tiRNA^Gly-GCC^ levels were significantly reduced following rotenone treatment (Fig. 2h), supporting the idea that ANG downregulation limits stress-induced tiRNA generation. These results are consistent with our observations in AD male brain samples.

### Aβ exposure downregulates ANG levels through oxidative stress-dependent mechanisms

Next, we investigated whether the sex-specific differences in ANG expression observed in AD postmortem brain tissue were driven by oligomeric Aβ. Following the hypothesis that oxidative stress reduces ANG levels at the molecular level, we would also expect Aβ to contribute to this reduction, resulting in lower levels of 5′-tiRNAs and, increased apoptosis, at least in the male model. To model Aβ burden, we employed two distinct approaches: utilizing a HEK293 APPwt AD cell model that exhibits an approximately 10-fold increase in APP-derived Aβ_1-40_ peptide levels or treating HEK293 control cells with oligomeric Aβ_1-42_ [53, 63]. Western blotting revealed significantly reduced ANG levels in APPwt cells compared to controls (Fig. 2i), suggesting that Aβ may contribute to ANG suppression. To confirm Aβ-specific effects, APPwt cells were treated with oligomeric Aβ_1-42_ (Fig. 2j) or the γ-secretase inhibitor DAPT (1 µM, 24 h, Fig. 2k), which blocks Aβ production (Supplementary Fig. S4 confirmed DAPT-induced reduction in Aβ levels). Following our hypothesis, oligomeric Aβ_1-42_ treatment further suppressed ANG protein levels (Fig. 2j), whereas DAPT restored the ANG amount to 73% of control levels (Fig. 2k), indicating that Aβ itself regulates ANG expression. Notably, vitamin C also rescued ANG levels in APPwt cells (Fig. 2l), reinforcing the role of Aβ-induced oxidative stress in ANG downregulation.

Given ANG’s established role in suppressing cytochrome c-mediated apoptosome formation through stress-induced cleavage of mature tRNAs into functional tRNA-derived fragments, we next investigated whether rANG treatment could attenuate caspase-3/7 activation in our AD model, followed by an assessment of its impact on 5′-tiRNA^Gly-GCC^ levels [34–36]. Caspase-3/7 activity was significantly elevated in HEK APPwt cells compared to controls (Fig. 2m), but was reduced following treatment with rANG (0.5 µg/mL for 2 h, Fig. 2n). Consistent with downregulated ANG protein levels, 5′-tiRNA^Gly-GCC^ levels were also significantly reduced in APPwt cells relative to controls (Fig. 2o). To assess whether the downregulation of 5′-tiRNA^Gly-GCC^ contributes to the loss of ANG’s protective effect in AD, we synthesized a 5′-tiRNA^Gly-GCC^ mimic and a complementary antisense inhibitor (Supplementary Table 1). Treatment of HEK APPwt cells with the tiRNA mimic significantly reduced caspase-3/7 activity, indicating an anti-apoptotic effect (Fig. 2p). Conversely, inhibition of endogenous 5′-tiRNA^Gly-GCC^ using the antisense oligonucleotide increased caspase activity, restoring apoptosis levels to those observed in untreated APPwt cells (Fig. 2q). Together, these findings demonstrate that 5′-tiRNA^Gly-GCC^ functions as a downstream effector of ANG’s cytoprotective activity, playing a key role in mitigating apoptosis under conditions of oxidative and amyloidogenic stress.

Collectively, these results from our HEK AD and rotenone-treated cell models align with our observations in male AD patients from the NBB, ADTBI, and ROSMAP cohorts, but not with those in female AD patients. The concurrent downregulation of ANG and 5′-tiRNA^Gly-GCC^ may contribute to increased neuronal vulnerability by impairing the cellular stress response, diminishing anti-apoptotic signaling, and ultimately compromising ANG’s protective functions in those individuals.

### Inflammasome-related inflammatory gene expression diverges between male and female AD patients and associates with ANG levels

Having linked ROS to reduced ANG protein levels, decreased 5′-^tiRNAGly-GCC^ abundance, and increased apoptosis, we next investigated the sex-specific ANG upregulation observed in female AD brains. To explain this contrast with ROS-mediated ANG suppression, we first explored hormonal influences on ANG, followed by the impact of inflammation.

**Fig. 3:**
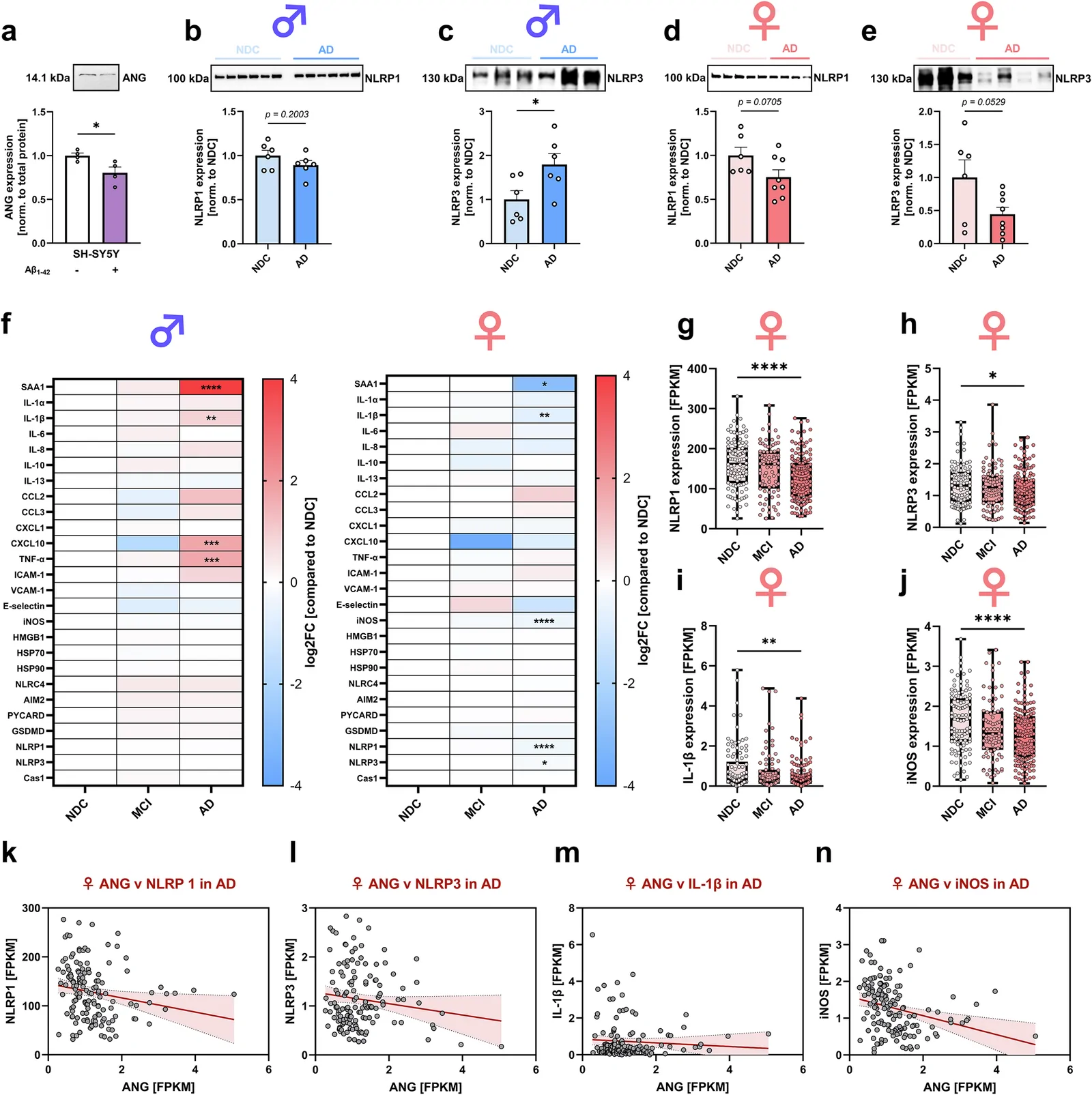
Inflammasome-related inflammatory gene expression diverges between male and female AD patients and associates with ANG levels in Alzheimer’s disease patients. **a** Western blot analysis of ANG protein levels in human neuronal SH-SY5Y cells treated with 100 nM oligomeric Aβ_1-42_ for 24 h. A significant downregulation of ANG was observed following treatment. Aβ-treated samples are compared to solvent-treated controls. Band intensities were normalized to the total amount of protein per sample. Data is shown as mean + SEM, *n* = 4 per group, Student’s unpaired t-test (*p < 0.05). **b** NLRP1 protein levels in postmortem cortex samples of male AD patients compared to controls, showing no significant difference. Band intensities were normalized to the total amount of protein per sample as loading control, quantified using Stain-Free™ technology (Bio-Rad Laboratories). Data is shown as mean + SEM, *n* = 6 per group, Student’s unpaired t-test. **c** NLRP3 expression in male AD cortex tissue, showing a significant upregulation compared to NDC. Band intensities were normalized to the total amount of protein per sample. Data is shown as mean + SEM, *n* = 6 per group, Student’s unpaired t-test (*p < 0.05). **d, e** NLRP1 and NLRP3 expression in female AD cortex tissue, showing a trend toward downregulation (NLRP1: *p* = 0.07505; NLRP3: *p* = 0.0529). Band intensities were normalized to the total amount of protein per sample. Data is shown as mean + SEM, *n* = 6–8 per group, Student’s unpaired t-test (*p < 0.05). **f** Heatmap of inflammation-related gene expression in the ROSMAP dataset from male AD patients, showing upregulation of key markers including NLRP1, NLRP3, IL-1β, and iNOS as well as female AD patients from the ROSMAP dataset, indicating a selective downregulation of inflammation markers. Log_2_FC relative to NDC individuals is shown with red indicating higher protein expression and blue lower protein expression. **g–j** Box plots showing significant downregulation of NLRP1 **g**, NLRP3 **h**, IL-1ß **i** and iNOS **j** in female AD patients from the ROSMAP cohort. Data is shown as mean + SEM, *n* = 83–142 per group, Student’s unpaired t-test (*p < 0.05, **p < 0.01, ****p < 0.0001). **k**–**n** Correlation analysis between ANG expression levels and inflammatory markers **k** NLRP1, **l** NLRP3, **m** IL-1β, and **n** iNOS in the female AD ROSMAP cohort. Higher ANG expression was associated with lower levels of each inflammatory marker, supporting an anti-inflammatory role of ANG in female AD patients. *n* = 83–142 per group. Uncropped images of Western blot membranes are provided in the Supplementary Information.

Oligomeric Aβ_1-42_ treatment significantly reduced ANG protein levels (Fig. 3a), consistent with findings in HEK-derived models. However,17β-estradiol treatment (alone or with Aβ_1-42_) also suppressed ANG expression (Supplementary Fig. S5a, b). This suggests estrogen signaling is unlikely to directly mediate ANG upregulation in female AD, which may instead reflect a compensatory response to neurodegeneration. Given ANG’s known role in suppressing inflammasome activation via tiRNA fragments, we examined whether elevated ANG and 5′-tiRNA^Gly-GCC^ levels in females were associated with reduced neuroinflammation, focusing on NLRP1 and NLRP3 as key markers [43]. We hypothesized an inverse correlation between ANG expression and NLRP activation in females. In male AD postmortem brain tissue, NLRP1 was unchanged while NLRP3 was significantly upregulated, suggesting increased inflammasome activity (Fig. 3b, c). In contrast, female AD patients showed a trend toward reduced NLRP1 and NLRP3 expression (p = 0.0750 and p = 0.0529; Fig. 3d, e). To validate this, we analyzed transcriptomic data from the larger ROSMAP cohort, examining NLRP1, NLRP3, IL-1β, and iNOS as additional inflammatory markers. Consistent with our tissue findings, male AD patients showed elevated expression of most inflammatory genes, while female AD patients had significantly lower levels of NLRP1, NLRP3, IL-1β, and iNOS (Fig. 3f–j). Correlation analyses further revealed a strong inverse association between ANG expression and these inflammatory markers in females, with individuals exhibiting high ANG levels showing markedly lower inflammatory gene expression than those with very low ANG levels, after controlling for sex and relevant covariates (Figs. 3k–n, S6). These findings support our hypothesis that ANG and its cleavage product 5′-tiRNA^Gly-GCC^ mediate anti-inflammatory effects in female AD patients, likely by suppressing the inflammasome and pro-inflammatory cytokines, thus contributing to neuroprotection. In contrast, increased oxidative stress in male AD patients appears to drive ANG downregulation, potentially impairing its protective function and promoting inflammation.

### ANG expression defines molecularly distinct subtypes within female AD patients

**Fig. 4:**
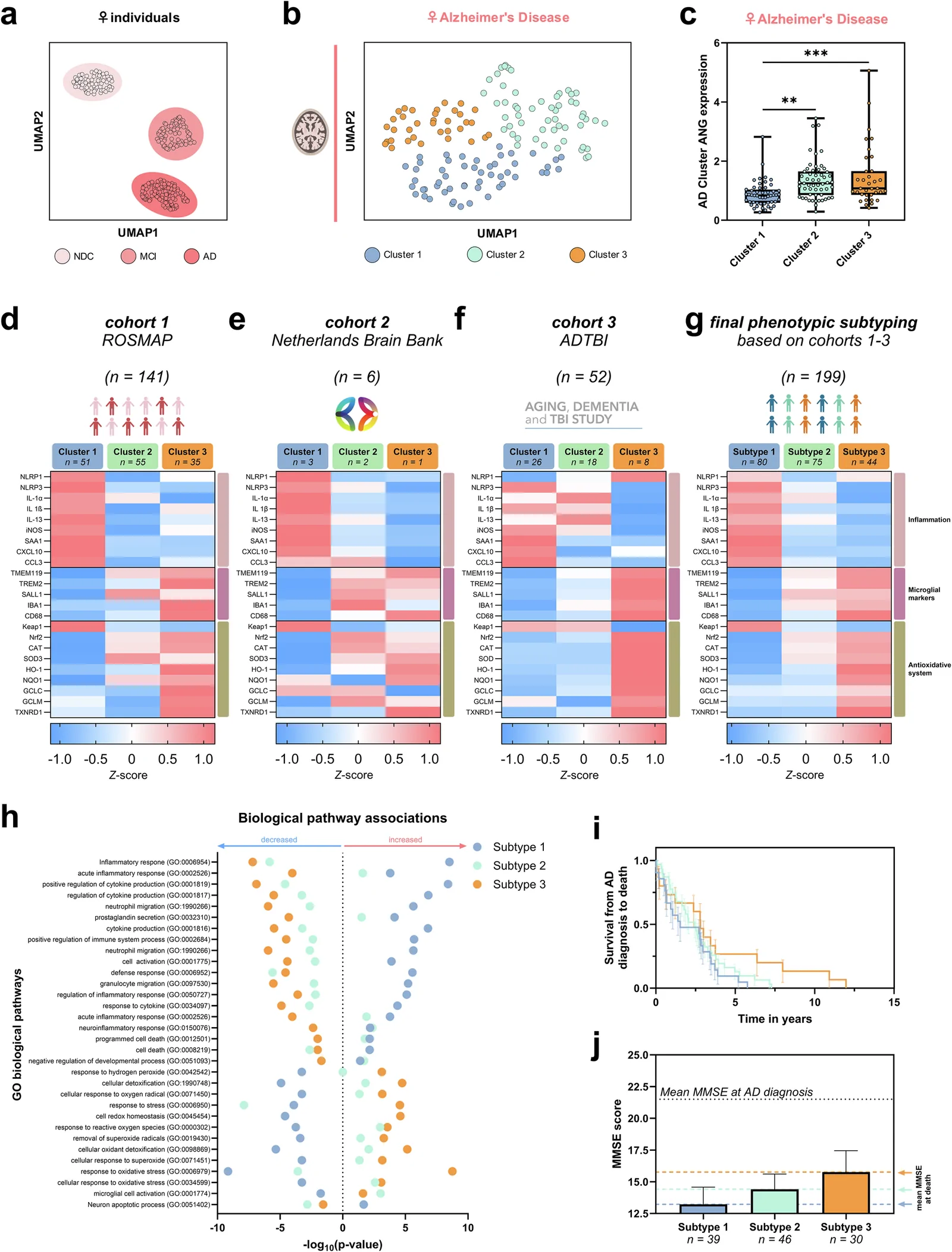
ANG stratifies female AD patients into subtypes with divergent survival time and cognitive decline. **a** Female NDC, MCI and AD patients projected to the uniform manifold approximation and projection (UMAP) space. **b** Female AD patients from the ROSMAP cohort projected to the uniform manifold approximation and projection (UMAP) space reveals three distinct transcriptomic clusters. **c** Box plot showing ANG expression levels across transcriptomic clusters. Cluster 1 exhibits the lowest ANG expression, whereas Cluster 3 shows the highest. *n* = 35–55 per group, ordinary one-way ANOVA, (***p < 0.001). **d** Protein mRNA levels (row) averaged across individuals within subtypes (columns). Cluster 0 differs in inflammatory response, microglial activation markers and antioxidant enzyme system gene expression compared to Cluster 2 with the highest ANG levels. **e** Replication of cluster building in the Netherlands brain bank cohort after mRNA sequencing. **f** Replication of clustering in cohort 3 from the Aging, Dementia and TBI study. **g** Final phenotypic subtyping combining data from all three cohorts together. **h** Gene Ontology (GO) biological pathways associated with the identified subtypes 1–3. Subtype 1 reveals increased neuronal death, inflammatory and oxidative stress pathways, whereas Subtype 3 showed a protective response. **i** Time from AD diagnosis to death within subtypes. *n* = 15–31 per group. The number of individuals included in this analysis differs from the original group sizes, as age at the time of AD diagnosis was not available for all patients. These individuals were excluded to allow an accurate assessment of survival time. **j** Clinical outcome using decline in MMSE scores from AD diagnosis to death according to subtypes. *n* = 30–46 per group. Individuals without documented MMSE scores at study inclusion, at the time of AD diagnosis, or prior to death were excluded from the analysis, as no comparison between the time of diagnosis and the last visit before death was possible for these patients.

Building on ANG’s correlation with reduced inflammation in female AD, we further characterized the female ROSMAP cohort to explore whether ANG expression defines biologically distinct subgroups. Using Uniform Manifold Approximation and Projection (UMAP) on transcriptomic data from NDC, MCI, and AD participants, we identified clear separation between groups, reflecting distinct molecular profiles (Fig. 4a). To assess intra-group heterogeneity, we applied UMAP with Hierarchical Density-Based Spatial Clustering of Applications with Noise (HDBSCAN) clustering. No subclusters were found in the NDC or MCI groups (Supplementary Fig. S7a, b), but three distinct clusters emerged within the female AD cohort (Fig. 4b), indicating molecular heterogeneity. ANG expression differed significantly across these clusters, with Cluster 1 showing the lowest levels (Mean_Cluster1_ = 0.89) and Clusters 2 and 3 showing progressively higher expression (Mean_Cluster2_ = 1.35, Mean_Cluster3_ = 1.47) (Fig. 4c). To determine if this effect was specific to the female AD cohort, we performed the same analysis in males. Interestingly, no clustering could be detected across the NDC, MCI, or AD groups in males using HDBSCAN (Supplementary Fig. S6c–f), suggesting a female-specific transcriptomic phenomenon.

### ANG-associated subtypes show divergent inflammatory and antioxidant signaling profiles

Next, we assessed whether transcriptomic differences across these ANG-based clusters correlated with key AD processes, such as inflammation and oxidative stress responses. We began by analyzing the expression of inflammatory genes, canonical microglial markers, and antioxidant enzymes using RNA-seq data from the ROSMAP study. Cluster 1, characterized by low ANG expression, exhibited robust upregulation of pro-inflammatory genes (NLRP1, NLRP3, IL-1β, iNOS, CXCL10), alongside elevated acute-phase proteins (SAA1) and chemokines (CCL3) (Fig. 4f). Conversely, Clusters 2 and 3, with higher ANG expression, showed attenuated inflammatory profiles. Similarly, homeostatic microglial markers (TMEM119, TREM2) were downregulated in Cluster 1 but relatively preserved in Clusters 2 and 3 (Fig. 4f). Genes involved in antioxidant defense (Nrf2, CAT, SOD, HO-1, NQO1, GCLC, GCLM, TXNRD1) also displayed markedly lower expression in Cluster 1, suggesting impaired oxidative stress response in low-ANG states. In contrast, these antioxidant genes were partially upregulated in Cluster 2 and strongly upregulated in Cluster 3, indicating enhanced redox homeostasis. Mechanistically, this activation of the antioxidant system is likely driven by increased Nrf2 expression, accompanied by a corresponding downregulation of the Nrf2 repressor Keap1 in Clusters 2 and 3. These patterns support a model where elevated ANG expression is associated with a neuroprotective transcriptional profile marked by reduced inflammation, preserved microglial homeostasis, and enhanced oxidative stress resilience.

To assess whether these subtypes are general or specific to female AD and ANG levels, we analyzed microglial and antioxidant marker expression across NDC, MCI, and AD groups in both sexes. Males showed no significant changes at any disease stage (Supplementary Fig. S7g–o). In females, most markers remained unchanged, except for TMEM119 and SALL1, which showed stage-specific expression (Supplementary Fig. S7p–x). Given this suggested specificity in female AD, we replicated the same clustering analysis in two independent cohorts: mRNA-seq data from postmortem cortical brain tissue (Cohort 2) and the ADTBI study (Cohort 3). Importantly, ANG expression levels across assigned clusters showed comparable patterns in all independent cohorts (Supplementary Fig. S8), consistent with the observations in the ROSMAP dataset. The cluster-specific expression patterns of inflammatory, microglial, and antioxidant markers were largely conserved across cohorts (Fig. 4g, h). This cross-cohort reproducibility led us to perform phenotypic subtyping using the combined dataset (*n* = 199). The resulting heatmap revealed three robust subtypes that mirrored the original clusters: Subtype 1 was characterized by high inflammation and low antioxidant signaling, while Subtype 3 showed reduced inflammation alongside upregulated antioxidant defenses. Subtype 2 represented an intermediate phenotype, with partial activation of microglial and antioxidant response pathways (Fig. 4i). This subtype-specific expression pattern suggests that ANG upregulation in female AD patients may result from enhanced antioxidant signaling, potentially linking Nrf2 activation to a more protective phenotype. To understand the biology of the identified AD subtypes, Gene Ontology (GO) biological pathway analysis in the female AD cohort revealed distinct subtype-specific associations (Fig. 4k). Subtype 1 was characterized by enriched inflammatory and immune responses, elevated oxidative stress, reduced antioxidant mechanisms, and increased neuronal apoptosis. Conversely, Subtype 3 displayed marked reductions in neuronal apoptotic signaling and robust activation of antioxidant defense pathways. Subtype 1 presented an intermediate profile, with pro-inflammatory pathways downregulated relative to Subtype 1, but less pronounced antioxidant enrichment than Subtype 3 (Fig. 4l), suggesting partial protective activation.

### ANG-associated female AD subtypes differ in survival and clinical outcomes

To assess whether the transcriptomic subtypes differed in clinical progression and overall outcome, we analyzed overall survival and longitudinal Mini-Mental State Examination (MMSE) scores in female AD patients. Individuals with missing clinical information (age at AD diagnosis) were excluded to ensure an accurate assessment of survival time. Subtype 1 exhibited the shortest average survival (1.5 years) following AD diagnosis. Subtype 2 had a moderately increased average survival with a hazard ratio (HR) of 0.78 (indicating a 22% lower instantaneous risk of death compared to Subtype 1); however, this difference was not statistically significant (*p* = 0.39). In contrast, Subtype 3 showed the longest average survival of 2.8 years, nearly double that of Subtype 1 (*p* = 0.04; Fig. 4l, Supplementary Table S4), with a significantly reduced hazard of death (HR = 0.47), corresponding to a 53% lower risk relative to Subtype 1.

Cognitively, Subtype 1 exhibited the strongest cognitive decline, reaching an average MMSE score of 13 at the last visit before death, with an average loss of 9 points. In contrast, Subtype 2 (average MMSE last visit=14; average loss 7 points) and Subtype 3 (average MMSE last visit=16; average loss 6 points) demonstrated slower cognitive decline, although the differences between the subtypes did not reach statistical significance (Fig. 4m; Supplementary Table S5). Only individuals with documented MMSE scores at study inclusion, at the time of AD diagnosis, and prior to death were included in the analysis to allow comparison across time points. Other clinical parameters (ApoE genotype, patient origin, postmortem delay, Braak stage) showed no significant correlations (Supplementary Fig. S9 and Tables S6–7). In contrast, analysis of key clinical markers in male AD patients revealed an average survival time of 1.58 years and a mean MMSE score of 13 at the time of death. These characteristics were comparable to those observed in female Subtype 1, which similarly displayed reduced ANG levels (Supplementary Fig. S10).

Collectively, these findings demonstrate that ANG expression stratifies female AD patients into biologically and clinically distinct subgroups. These subtypes exhibit divergent inflammatory and antioxidative profiles, microglial activation states, survival rates, and rates of cognitive decline.

## Discussion

In this study, we investigated sex-dependent regulation of ANG and its tRNA-derived fragments in Alzheimer’s disease, revealing reduced mRNA and protein levels in male AD patients and increased mRNA and protein levels in female AD patients. Our analyses indicated a phenotype in males that is consistent with increased oxidative stress signaling, while in females, we identified three distinct molecular subtypes characterized by divergent inflammatory responses, oxidative stress pathways, microglial activation, and clinical outcomes, including differences in survival and cognitive decline. Based on this, we propose a functional classification of the female subtypes: Subtype 1 might represent a neuroinflammatory profile with increased neuronal apoptotic processes, sharing similarities with the male phenotype. Subtype 2 appears to be an intermediate, partially protective subtype with a distinct microglial activation pattern. In contrast, Subtype 3 appears to display a protective, homeostatic signature, characterized by active microglial phagocytic engagement and extended overall survival.

The observed sex-specific dysregulation of ANG mRNA and protein expression in our experiments constrained by post mortem sample size aligns with prior, although limited, studies. Kim et al. reported lower serum ANG in a predominantly male cohort, while Wu et al. observed increased ANG mRNA levels in a mostly female cohort [66, 67]. These concordant trends across cross-sectional datasets suggest that sex-specific differences in ANG mRNA expression contribute to the observed protein-level changes, potentially further shaped by post-transcriptional regulation. However, these findings should be interpreted as associative rather than causal. Additional support for sex-linked molecular heterogeneity in AD comes from a recent study by Seto et al., who identified more than 2000 proteins whose associations with β-amyloid, tau pathology, and longitudinal cognitive decline differed by sex, many mapping to sex-chromosome–linked regulatory networks [68]. These findings further underline the hypothesis that AD pathobiology is shaped by sex-dependent molecular programs, consistent with the ANG-related divergence observed in our study. To elucidate the molecular mechanisms underlying ANG dysregulation in both sexes, we employed cellular models of AD. Our findings suggest that ANG and 5′-tiRNA^Gly-GCC^ downregulation are modulated by oxidative stress, as their expression was suppressed by rotenone and Aβ, and restored by antioxidant treatment or γ-secretase inhibition. These results are consistent with ROS being linked to changes in ANG expression, particularly in the male phenotype. This hypothesis is supported by findings in α-synuclein–overexpressing cells, where toxic α-synuclein aggregates, similar to Aβ, disrupt mitochondrial complex I, elevate ROS production, and downregulate ANG expression [27, 69–71]. Furthermore, the protective effects of recombinant ANG observed in our models, including reduced caspase-3/7 activity, may reflect an association between ANG signaling and cellular resilience, potentially involving the Nrf2 pathway and reduced oxidative stress, consistent with previous reports [36, 39]. Building on ANG’s role in apoptosis regulation, we further examined its downstream effector, 5′-tiRNA^Gly-GCC^. Our results suggest that this fragment modulates stress-induced apoptosis, aligning with previous studies that support a cooperative model where ANG and 5′-tiRNA^Gly-GCC^ promote cell survival by limiting mitochondrial dysfunction and apoptotic signaling under oxidative stress [72, 73]. Collectively, these data raise the possibility that oxidative stress–associated downregulation of ANG may coincide with alterations in cellular defense pathways, potentially contributing to increased vulnerability to neurodegenerative stressors [34]. However, as oxidative stress alone did not account for ANG upregulation in female AD, we next investigated its potential involvement in inflammatory signaling, particularly via the NLRP3 inflammasome. ANG-derived tiRNAs are known to suppress NLRP3 activation, and consistent with this, our study found that elevated ANG expression was associated with reduced NLRP3 and other inflammatory genes in a subset of female AD patients [43]. This observation aligns with findings by McManus et al., who identified NLRP3 as a key driver of chronic neuroinflammation and cognitive decline in AD, with its inhibition enhancing microglial phagocytosis, mitochondrial function, and promoting a distinct microglial phenotype linked to improved Aβ clearance and mitochondrial signaling [41, 42]. These observations raise the possibility that ANG mRNA and protein upregulation in females may be linked to modulation of inflammatory pathways. Since ANG mRNA and protein levels are altered in a similar direction, the primary regulatory mechanism cannot be inferred from steady-state measurements alone. Coordinated changes at both levels may arise from transcriptional regulation, altered mRNA stability, differences in translation efficiency, or changes in protein turnover, as well as upstream factors affecting multiple layers of gene expression. Future studies combining analyses of nascent transcription, mRNA decay, translation efficiency, and protein stability will be required to determine the principal regulatory mechanisms underlying ANG dysregulation. Although acute 17β-estradiol exposure in our experimental system did not support a simple direct induction of ANG, this finding should be interpreted cautiously within the broader and multifactorial context of sex-hormone biology in AD. Estrogenic regulation in the aging female brain is multifaceted and extends far beyond a single ligand exposure paradigm, encompassing chronic menopausal hormone decline, receptor subtype–specific signaling, local neurosteroid metabolism, and interactions with other endocrine pathways. Consequently, broader estrogenic or sex-hormonal contributions to ANG dysregulation in female AD cannot be excluded. Rather, the female-specific ANG increase observed here may arise from a complex interplay between neurodegenerative stress responses and sex-dependent hormonal remodeling during disease progression. Further studies are required to fully elucidate these mechanisms. Independently, our identified female AD molecular subtypes (neuroinflammation, intermediate, and protective/homeostatic) align closely with emerging subtype frameworks, such as the cerebrospinal fluid (CSF) proteomic subtypes described by Tijms et al. which are similarly associated with distinct genetic risk profiles and transcriptomic signatures including neuronal hyperplasticity, innate immune activation, and blood-brain barrier (BBB) dysfunction [74]. Subtype 1, characterized by the lowest ANG expression and elevated inflammatory gene levels, appears to parallel the *‘Innate Immune Activation’* CSF subtype, marked by a pronounced pro-inflammatory state [74]. In contrast, Subtype 3, defined by high ANG expression, reduced inflammation, upregulation of homeostatic microglial markers, Nrf2 signaling, and oxidative stress response genes, may reflect a more protective phenotype with enhanced cellular stress resilience. While Subtype 3 does not perfectly mirror any single CSF proteomic subtype, its profile suggests a nuanced immune state potentially overlapping with the *‘Neuronal* Hyperplasticity’ subtype or a milder form of *‘Innate Immune Activation’* where homeostatic microglia support synaptic remodeling and regulated neuroimmune responses [74]. Subtype 3 was associated with prolonged survival and slower cognitive decline compared to the steeper decline observed in Subtype 1. Although distinct clustering could not be determined in the male AD cohort, the overall group exhibited survival rates and MMSE scores comparable to those of female Subtype 1, suggesting the greatest similarity with this subtype. This is further supported by the markedly reduced ANG levels observed in male AD patients. These sex-divergent patterns suggest that ANG-related stress-response pathways are organized differently in males and females with AD, which may explain why ANG-associated molecular subtypes, including the neuroprotective Subtype 3, emerge only in the female cohort. In contrast, the relative uniformity of ANG suppression together with heightened inflammatory signaling in males may coincide with lower transcriptional variability, which could make ANG-defined subgroups more difficult to detect. This hypothesis is supported by recent multi-omics studies demonstrating widespread sex-dependent gene–phenotype associations in AD, including thousands of proteins whose relationships with amyloid, tau, and cognition differ by sex and map to sex-linked regulatory networks, collectively indicating greater molecular heterogeneity in females [68]. Consistent with this, López-Cerdán et al. further reported that sex-based molecular differences are more pronounced in female AD patients, including proteins with significantly distinct activity patterns compared with males [75]. Together, these findings suggest a model in which females may exhibit greater molecular heterogeneity and, in a subset of patients, maintain the potential for ANG-mediated compensatory responses, whereas males appear to show a more uniform, ANG-suppressed pro-inflammatory profile. Although the identification of the neuroprotective Subtype 3 might initially appear contradictory to the higher lifetime risk, faster cognitive decline, and greater neuropathological burden observed in women, these findings align with emerging evidence that females exhibit greater neural resilience to brain aging earlier in the disease course [76]. Females appear able to initially maintain more cognitive performance and hippocampal volume for a given burden of amyloid-β than males, suggesting a capacity to tolerate accumulating AD pathology while sustaining compensatory mechanisms over an extended period [76]. Once a critical threshold is exceeded, this resilience may collapse after years of compensation, leading to accelerated cognitive decline and rapid hippocampal atrophy after diagnosis [75, 76]. Within this framework, ANG-associated neuroprotective Subtype 3 may represent a molecular key signature of preserved compensatory capacity in a subset of females, in which ANG-dependent signaling might support a homeostatic, anti-inflammatory state under disease pressure until resilience mechanisms ultimately fail. Supporting this hypothesis, several experimental studies provide functional evidence that ANG directly suppresses inflammatory signaling. In human corneal fibroblasts, ANG treatment reduces TNF-α– or LPS-induced expression of the pro-inflammatory cytokines IL-1β, IL-6, and IL-8, cytokines that are also reduced in high-ANG S3 female AD patients in our dataset, and downregulates NF-κB activation, while increasing the anti-inflammatory cytokines IL-4 and IL-10, thereby significantly attenuating inflammation [77, 78]. In rodent models of immune-mediated ocular inflammation, ANG administration similarly reduced inflammatory cell infiltration, and expression of IL-1β, IL-8, TNF-α, iNOS, and NF-κB [79]. This ANG-dependent anti-inflammatory effect may also help explain the absence of comparable transcriptomic phenotypes in males, whose brains appear less able to engage the same anti-inflammatory compensation programs [76]. Our findings suggest that ANG-targeted therapeutic strategies may need to be applied in a sex- and subtype-specific manner rather than uniformly across all AD patients. Emerging evidence supports such a personalized approach in AD, where sex-dependent differences shape pathological pathways and influence therapeutic responsiveness [13, 68, 80, 81]. In particular, the neuroprotective phenotype observed in female Subtype 3 raises the possibility that elevated ANG expression may reflect a compensatory response, potentially linked to antioxidant and anti-inflammatory signaling. In this context, further augmentation of ANG signaling may not provide additional benefit and could potentially disrupt existing homeostatic and compensatory mechanisms. Moreover, given the known pro-proliferative effects of ANG via PI3K/Akt and ERK1/2 signaling pathways, including induction of factors such as TGF-β1 that are associated with cell migration and tumor progression, excessive ANG activation may raise safety concerns, particularly in non-neuronal contexts [82–84]. Conversely, patients with suppressed ANG expression, such as males or females within the neuroinflammatory Subtype 1, may benefit more from strategies aimed at restoring ANG activity or increasing protein levels. However, these groups are characterized by distinct regulatory environments, including elevated oxidative stress and inflammatory signaling, which may influence the downstream effects of ANG activation and limit its efficacy if not addressed concurrently. Accordingly, combination strategies targeting both ANG restoration and inflammatory or oxidative stress pathways may be required. In line with this, ROS-sensitive ANG conjugates have been proposed as a promising approach for context-dependent activation under oxidative stress conditions; however, their efficacy and safety in blood–brain barrier models remain to be established [85]. Delivering ANG safely and effectively to the brain remains a major challenge, requiring further development of BBB-permeable variants, nanotechnology-based delivery systems, and ROS-responsive dosing strategies [40]. Overall, given the pleiotropic nature of ANG signaling, ANG-targeted interventions may exert divergent effects depending on the biological context and therefore require careful patient stratification and context-dependent validation before clinical translation.

Despite the multi-cohort transcriptomic integration and mechanistic analyses presented here, several limitations remain. Sample sizes within individual subgroups are modest, particularly for survival and cognitive analyses, and these findings should therefore be interpreted as hypothesis-generating. While our data link ANG and the generation of 5′-tiRNA^Gly-GCC^ to oxidative stress, inflammasome signaling, and apoptotic pathways, they do not yet establish ANG as a causal driver and may instead reflect broader inflammatory and oxidative stress programs in AD. In addition, the mechanistic validation in HEK293-based APP cell models must be interpreted with caution, as these non-neuronal cells do not fully recapitulate the neuronal transcriptomic landscape, RNA metabolism, amyloid stress responses, or neuron–glia interactions characteristic of the human brain. This is particularly relevant for ANG-dependent tRNA cleavage and downstream tiRNA generation, which may be strongly cell-type specific. Nevertheless, the observed ANG regulation was reproduced in the neuronal SH-SY5Y model, supporting that the core expression phenotype is not restricted to the HEK293 background, although its precise translation to human neuronal and glial biology remains to be established. Furthermore, the subtype definitions are currently based on bulk postmortem transcriptomic datasets and therefore cannot resolve the precise cellular origin or anatomical context of the observed signatures. Although the postmortem interval was short and tightly controlled across samples (3–6 h), residual confounding from agonal factors, including terminal hypoxia, systemic inflammation, and premortem physiological stress, cannot be fully excluded, as these variables may directly influence ANG expression and tiRNA generation. In addition, residual regional and cellular heterogeneity within the sampled tissue, as well as premortem medication exposure, particularly cholinesterase inhibitors, antidepressants, antipsychotics, sedatives, or anti-inflammatory drugs, may modulate stress, inflammatory, and RNA-processing pathways relevant to ANG biology. However, detailed clinical metadata on prior medication use were not available for the analyzed samples.

Future studies employing single-cell and spatial transcriptomics, together with in vivo manipulation of ANG signaling, will be required to validate these subtype structures and to more directly examine potential causal mechanisms. Nevertheless, the integration of mechanistic experiments with multi-cohort human transcriptomics is consistent with the possibility that ANG-associated RNA stress pathways may contribute to the molecular heterogeneity underlying Alzheimer’s disease. By linking ANG-related RNA stress signatures to oxidative and inflammatory pathways across experimental systems and human cohorts, our findings suggest a conceptual framework for understanding sex- and subtype-specific vulnerability in Alzheimer’s disease and underscore the potential importance of stratified approaches in future mechanistic and therapeutic investigations.

## Supplementary information

Supplementary Figures

Supplementary Information and Methods

## Data Availability

Data from the Aging, Dementia, and Traumatic Brain Injury (ADTBI) Study are publicly available at http://aging.brain-map.org/download/index and require no additional access. RNA-seq data from the ROSMAP study were retrieved from Synapse (accession: syn23446022) and are available under controlled access in accordance with human subject privacy regulations. Researchers must complete a data use agreement, required solely to protect the anonymity of ROSMAP participants. This agreement can be arranged either through Rush University Medical Center (RUMC) or through SAGE, which hosts the Synapse.org platform, where the relevant documentation is provided. All other data are included in the Supplementary information or available from the corresponding author upon reasonable request.
