# Supplementary material for: Sex-specific regulation of angiogenin in Alzheimer’s disease
Source: Mol Psychiatry. 2026 Jun 2;31(10):5735–48. doi: 10.1038/s41380-026-03656-7 (PMC13569426; doi:10.1038/s41380-026-03656-7)
Supplement: Supplementary file 1 [file 41380_2026_3656_MOESM1_ESM.docx]

**Sex-specific regulation of Angiogenin in Alzheimer’s Disease**

Marko Jörg^1*^, Lukas Walz^1^, Sebastian Nathal^1^, Marco Kristen^1^, Christine Lietz^1^, Max Müller^1^, Vu Thu Thuy Nguyen^2^, Nicolas Ruffini^3^, Marie-Luise Winz^1^, Susanne Gerber^3^, Kristina Endres^2,4^, Mark Helm^1^, Kristina Friedland^1*^

^1^Institute of Pharmaceutical and Biomedical Sciences, Johannes Gutenberg University, Mainz, Germany

^2^Department of Psychiatry and Psychotherapy, University Medical Center of the Johannes Gutenberg University, Mainz, Germany

^3^Institute of Human Genetics, University Medical Center Johannes Gutenberg University, Mainz, Germany

^4^Campus Zweibrücken, University of Applied Sciences Kaiserslautern, Zweibrücken, Germany

* To whom correspondence should be addressed. Tel: +49 6131 39 20337; Email: [kfriedla@uni-mainz.de](mailto:kfriedla@uni-mainz.de), Tel: +49 6131 39 23236, [marjoerg@uni-mainz.de](mailto:marjoerg@uni-mainz.de)

**SUPPLEMENTARY DATA AND FIGURES**

**Supplementary Figures**

**Supplementary Fig. S1: Validation of ANG antibody specificity and sex-dependent differences in ANG expression at the protein and mRNA levels in Alzheimer’s disease. a** Western blot showing ANG protein detection from murine and human source upon pre-incubation with (+) or without (-) ANG-specific blocking peptide. Pre-incubation with the blocking peptide effectively reduces the ANG-specific signal at around 14-17 kDa, confirming antibody specificity at this height. Lane 1 ANG standard protein. Lane 2-5 protein lysate from mouse liver. Lane 6-9 HEK293 control protein lysates. GAPDH is shown as a loading control. **b** Direct comparison of ANG protein levels between male and female AD patients on the same Western blot membrane revealed distinct sex-dependent patterns of ANG expression. *n* = 6-8 per group; unpaired Student’s t-test; (****p* < 0.001). **c-d** ANG mRNA expression was compared between male and female AD patients in the ROSMAP (c) and ADTBI (d) cohorts. *n* = 13-144 per group; unpaired Student’s t-test; (****p* < 0.001).

**Supplementary Fig. S2: ANG protein alterations in 5xFAD AD animal model in cortex and peripheral organs. a** Western blot analysis showed no significant change in cortical brain samples of 5xFAD AD male and female mice at the age of 9-12 weeks compared to respective WT control. **b** Representative Western blot and bar graph showing ANG protein expression to be decreased in the cortex of male 5xFAD AD mice (blue) and increased in female 5xFAD AD mice (light red) at the age of 42-55 weeks compared to their respective WT control. (*n* = 3-4 for male/female 5xFAD AD mice and *n* = 3-8 for control WT mice, Student’s unpaired t-test, (****p* < 0.001). **c-f** Expression of ANG in mouse tissue in both sex at different age stages: Expression of ANG in mouse (c) liver tissue, (d) lung tissue, (e) kidney tissue, and (f) spleen tissue, showing no age- and sex-specific variations, comparing WT and TG mice. Statistical analysis: *n* = 2-9 per group; data analyzed using ordinary one-way ANOVA followed by Tukey's multiple comparisons test. No significant changes were detected between groups. Abbreviations: m = male, f = female, y = young, o = old, WT = wildtype, TG = transgenic.

**Supplementary Fig. S3: ANG protein expression in rotenone-treated HEK293 control cells.** Western blot analysis showing ANG protein levels following treatment with 5 µM rotenone for 1 h, 6 h, and 24 h. A significant reduction in ANG expression was observed at 6 h and 24 h, while 1 h of treatment showed no significant change. Protein levels were normalized to β-actin. *n* = 6-9 per group; unpaired Student’s t-test; (****p* < 0.001).

**Supplementary Fig. S4: Human Aβ_1-40_ levels in HEK293 control and APPwt cells. A** Levels of Aß_1-40_ after determination with ELISA kit show significantly increased levels for APPwt. After DAPT treatment APPwt cells show significantly reduced human Aβ_1-40_ levels compared to untreated control. *n* = 3-4, Student’s unpaired t-test, (*****p* < 0.0001).

**Supplementary Fig. S5: Expression of ANG and ERα in SH-SY5Y cells treated with 17β-Estradiol and oligomeric Aβ_1-42_. a** Expression of estrogen receptor alpha (ERα) in response to 17β-Estradiol, oligomeric Aβ_1-42_, or their combination. Treatment with 17β-Estradiol significantly increased ERα levels. Band intensities were normalized to the total amount of protein per sample as loading control, quantified using Stain-Free™ technology (Bio-Rad Laboratories). **b** ANG protein expression levels in SH-SY5Y cells treated with oligomeric Aβ_1-42,_ 17β-Estradiol, or a combination of both. Western blot analysis revealed that both oligomeric Aβ_1-42_ and 17β-Estradiol significantly reduced ANG expression. Band intensities were normalized to the total amount of protein per sample as loading control, quantified using Stain-Free™ technology (Bio-Rad Laboratories). *n* = 4 per group. Protein expression was normalized to total protein. Relative expression is shown as fold change compared to solvent-treated controls. Student’s unpaired t-test; (**p* < 0.05).

**Supplementary Fig. S6. Expression of inflammatory, inflammasome, and microglial markers as well as oxidative stress response enzymes in male and female NDC, MCI, and AD individuals. a** Comparison of IL-6 and IL-8 levels between individuals with extremely high and low ANG expression. Two-way-ANOVA followed by Tukey’s multiple comparison test (**p* < 0.05, *****p* < 0.0001). **b-h** mRNA expression of key molecules NLRP1 (b), NLRP3 (c), IL-1ß (d), IL-6 (e), IL-8 (f), iNOS (g) and Nrf2 (h) was compared between male and female AD patients in the ROSMAP cohort. Student’s unpaired t-test; (**p* < 0.05).

**Supplementary Fig. S7. Expression of inflammatory, inflammasome, and microglial markers as well as oxidative stress response enzymes in male and female NDC, MCI, and AD individuals. a-b** UMAP projections of transcriptomic profiles from female NDC (a) and MCI (b) individuals. **c** UMAP projection of male NDC, MCI, and AD patients. **d-f** UMAP projections of transcriptomic data from male NDC (d), MCI (e), and AD (f) individuals show no distinct clustering. **g-k** Expression of microglial activation markers in male NDC, MCI, and AD individuals from the ROSMAP cohort. **l-o** Expression of antioxidant response enzymes in male NDC, MCI, and AD individuals. **p-t** Expression of microglial activation markers in female NDC, MCI, and AD individuals from the ROSMAP cohort. **u-x** Expression of antioxidant response enzymes in female NDC, MCI, and AD individuals. *n* = 71-142 per group. Statistical analysis was performed using ordinary one-way ANOVA followed by Tukey's multiple comparisons test.

**Supplementary Fig. S8. Replication of data-driven clustering in independent cohorts. a** Female AD patients from Cohort 2 projected into Uniform Manifold Approximation and Projection (UMAP) space revealed three distinct transcriptomic clusters using the same analytical pipeline as in Cohort 1. Box plots show ANG expression levels across clusters, with Cluster 1 exhibiting the lowest and Cluster 3 the highest ANG expression. n = 1-3 per group; ordinary one-way ANOVA (***p < 0.001). **b** The analysis was replicated in an independent cohort from the ADTBI study, revealing comparable clustering patterns and ANG expression differences across subtypes. **c** Sensitivity analyses indicated that lowering cluster_selection_epsilon or min_cluster_size increased cluster granularity, resulting in additional microclusters as expected. Notably, these additional clusters represented subdivisions within the originally defined states rather than a change in the overarching subtype architecture, as samples continued to segregate into groups with consistently low versus high ANG expression. n = 8-26 per group; ordinary one-way ANOVA (*p < 0.05, **p < 0.01, ***p < 0.001).

**Supplementary Fig. S9. Clinical information and parameters across subtypes. a-c** ApoE gene distribution across subtypes. **d-e** Ethnicity distribution across subtypes 1-3 **g-i** Spanish, Hispanic or Latino origin distribution within subtypes 1-3. For panels d-i, naming and classification were defined by the ROSMAP study and not by the authors. **j** Braak stages across different subtypes. **k** Postmortem delay across subtypes. **l** Years of education across subtypes 1-3. j-l Statistical analysis was performed using ordinary one-way ANOVA followed by Tukey's multiple comparisons test.

**Supplementary Fig. S10. Clinical parameters in male patients. a** Survival rates of male AD patients are shown; the median survival time from AD diagnosis to death was 1.58 years. **b** MMSE scores at the time of death across male NDC, MCI, and AD patients are shown; the mean MMSE score in the AD group was 13.48. Statistical analysis was performed using ordinary one-way ANOVA followed by Dunnett's multiple comparisons test (*p <0.05, ****p < 0.0001). **c** Braak stages in male NDC, MCI, and AD patients indicate a significant increase in Braak stages in AD patients compared to NDC. Statistical analysis was performed using ordinary one-way ANOVA followed by Dunnett's multiple comparisons test (**p* < 0.05, *****p*  < 0.0001). **d** Years of education in male NDC, MCI, and AD patients are also shown.
