# Supplementary material for: Sex-specific regulation of angiogenin in Alzheimer’s disease
Source: Mol Psychiatry. 2026 Jun 2;31(10):5735–48. doi: 10.1038/s41380-026-03656-7 (PMC13569426; doi:10.1038/s41380-026-03656-7)
Supplement: Supplementary file 2 [file 41380_2026_3656_MOESM2_ESM.docx]

**Sex-specific regulation of Angiogenin in Alzheimer’s Disease**

Marko Jörg^1*^, Lukas Walz^1^, Sebastian Nathal^1^, Marco Kristen^1^, Christine Lietz^1^, Max Müller^1^, Vu Thu Thuy Nguyen^2^, Nicolas Ruffini^3^, Marie-Luise Winz^1^, Susanne Gerber^3^, Kristina Endres^2,4^, Mark Helm^1^, Kristina Friedland^1*^

^1^Institute of Pharmaceutical and Biomedical Sciences, Johannes Gutenberg University, Mainz, Germany

^2^Department of Psychiatry and Psychotherapy, University Medical Center of the Johannes Gutenberg University, Mainz, Germany

^3^Institute of Human Genetics, University Medical Center Johannes Gutenberg University, Mainz, Germany

^4^Campus Zweibrücken, University of Applied Sciences Kaiserslautern, Zweibrücken, Germany

* To whom correspondence should be addressed. Tel: +49 6131 39 20337; Email: [kfriedla@uni-mainz.de](mailto:kfriedla@uni-mainz.de), Tel: +49 6131 39 23236, [marjoerg@uni-mainz.de](mailto:marjoerg@uni-mainz.de)

**SUPPLEMENTARY INFORMATION**

**Supplementary Methods for experimental procedures described in the main manuscript**

*Human postmortem cortical brain samples*

All human postmortem brain samples were obtained from the Netherlands Brain Bank (NBB, Amsterdam, The Netherlands) under project number 1341. Ethical guidelines and approval for autopsy and brain area donation are outlined in the Ethical and Legal Declaration of the Netherlands Brain Bank. All autopsies were performed by NBB itself at the VU University Medical Center (Amsterdam, the Netherlands) with approval from the institutional ethics committee. All samples from demented individuals were clinically and neuropathologically confirmed cases of AD, as verified by the NBB using established biochemical and histopathological methods. Clinical diagnoses were based on the criteria of the Consortium to Establish a Registry for Alzheimer’s Disease (CERAD). Non-demented controls exhibited no clinical symptoms or pathological features of dementia at the time of death. For analysis, tissue from the superior frontal gyrus (Brodmann areas gfs 3 and gfs 4) was selected due to its relevance in AD pathology.

*Human databases*

Data from the Aging, Dementia, and Traumatic Brain Injury (ADTBI) Study (primary publication: Miller J.A. et al.) were retrieved from <http://aging.brain-map.org/download/index> on January 20, 2020 [1]. Primary extraction of both normalized and non-normalized fragments per kilobase per million (FPKM) values was performed using a custom R script (R version 3.6.0, “Planting of a Tree”; R Core Team, 2018) and saved in an Excel file (.xlsx). Normalized FPKM values for ANG, corrected for RNA integrity number (RIN) and batch effects, were used for analysis. Only cortical samples from male patients and female patients without traumatic brain injury (TBI) history were included. To enable cross-study comparison, expression values for AD samples were normalized to the NDC cohort. In addition, RNA-seq data from the ROSMAP study were retrieved from Synapse (accession: [syn23446022](https://www.synapse.org/Synapse:syn23446022)) following approved data access. Normalized FPKM values were analyzed from cortical tissue samples representing three diagnostic groups: NDC, individuals with MCI, and clinically diagnosed AD. This stratification enabled evaluation of gene expression changes across the cognitive decline spectrum. Only cortical samples were included to ensure tissue-type consistency and relevance to AD pathogenesis. This stratification enabled evaluation of gene expression changes across the cognitive decline spectrum.

*Cell culture*

Human embryonic kidney (HEK293) cells were stably transfected with a pcDNA3.1(+) construct encoding wild-type amyloid precursor protein (APPwt) under the control of a cytomegalovirus (CMV) promoter, using FuGENE® 6 transfection reagent [2, 3]. These transgenic cell line overexpress APPwt and produce distinct levels of Aβ_1-40_ and Aβ_1-42_ peptides [2, 3]. Untransfected HEK293 cells (HEK293 ut, used as controls) were maintained in Dulbecco’s Modified Eagle Medium (DMEM; Gibco™, Cat. No. 41965-039) supplemented with 10% heat-inactivated fetal calf serum (FCS; Gibco™, Cat. No. 10270-106, Lot: 2156880) and 50 U/mL penicillin-streptomycin (Gibco™, Cat. No. 15140-122). APPwt cells were cultured in the same medium with the addition of 400 µg/mL Geneticin (G418; Roche, Cat. No. 4727894001) for selection. All cells were incubated at 37 °C with 5% CO₂ in a humidified atmosphere.

To assess sex-specific effects, SH-SY5Y human neuroblastoma cells were used. Cells were maintained in high-glucose DMEM (Gibco™, Cat. No. 10569010) supplemented with GlutaMAX™, 10% heat-inactivated fetal bovine serum (FBS; Gibco™, Cat. No. 10270-106), 60 U/mL penicillin and 60 µg/mL streptomycin (Gibco™, Cat. No. 15140-122), 1% MEM vitamin solution (100×; Gibco™, Cat. No. 11120037), 1% MEM non-essential amino acids (100×; Gibco™, Cat. No. 11140035), and 1 mM sodium pyruvate (Thermo Scientific™, Cat. No. 11360039). Cells were cultured under identical incubation conditions (37 °C, 5% CO_2_).

*Aβ_1-42_ Oligomerization*

To analyze the effects of stressors on *ANG* expression, HEK293 control were treated with 100 nM oligomeric Aβ_1-42_ (AggreSure β-Amyloid (1-42), AnaSpec, AS-72216) for 24 hours. Aβ_1-42_ oligomers were prepared according to the manufacturer's instructions by incubating the peptide at 4 °C for 24 hours to promote oligomerization. Successful aggregation was verified using Thioflavin T staining (AnaSpec, AS-88306) with fluorescence measured at 484 nm excitation and 440 nm emission [4].

*Preparation of protein extracts*

To analyze ANG expression during AD conditions, 1 × 10^6^ HEK293 control cells were seeded in 100 mm dishes 24 hours prior to treatment. For mitochondrial complex I inhibition, cells were treated with 5 µM rotenone for 6 hours. To evaluate the effect of vitamin C (Sigma-Aldrich®, Cat. No. SKU: A92902), cells were treated with 1 mM vitamin C (pH adjusted to 7.4) for 24 hours. In a sequential treatment experiment, cells were pretreated with 5 µM rotenone for 6 hours, followed by 1 mM vitamin C for 24 hours to assess recovery of ANG expression. For γ-secretase inhibition, cells were treated with 1 µM DAPT (Sigma-Aldrich®, Cat. No. D5942) for 24 hours. HEK293 cells were included as controls. To confirm the effect of DAPT on Aβ production, Aβ_1-40_ levels were measured using the Amyloid Beta 40 Human ELISA Kit (Invitrogen, Cat. No. KHB3482) according to the manufacturer’s protocol. Supernatants were collected after 24 hours from DAPT-treated and untreated cells. For antioxidant rescue, HEK APPwt cells were treated with 1 mM vitamin C (pH 7.4) for 24 hours. Additionally, cells were treated with oligomeric Aβ for 24 hours (AggreSure, Anaspec, Cat. No. AS-72216), with oligomerization carried out following the manufacturer's protocol. For vitamin C treatment, HEK APPwt cells were treated with 1 mM Vitamin C, adjusted to pH 7.4, for 24 hours.

For Western blot analysis, culture medium was removed and cells were washed twice with ice-cold PBS. Cells were lysed in 200 µL of radioimmunoprecipitation assay (RIPA) buffer containing: 50 mM Tris-HCl (pH 7.4; Carl Roth, Cat. No. 5429.5), 150 mM NaCl (Carl Roth, Cat. No. P029.3), 1% Triton™ X-100 (Carl Roth, Cat. No. 3051.4), 0.5% sodium deoxycholate (Sigma-Aldrich®, Cat. No. 30970), 0.1% SDS (Carl Roth, Cat. No. CN30.2), 5 mM EDTA (Carl Roth, Cat. No. CN06.2), and 1 mM PMSF (Carl Roth, Cat. No. 6367.1). Lysates (from both cells and tissues) were incubated for 45 minutes at room temperature and centrifuged at 10,000 × g for 10 minutes at 4 °C. Supernatants containing soluble proteins were collected, and total protein concentration was determined using the Bradford assay (Bio-Rad Protein Assay Dye Reagent Concentrate; Bio-Rad, Cat. No. 5000006) according to the manufacturer’s instructions.

*RNA Extraction from cultured cells and postmortem brain tissue*

Total RNA was extracted from cultured cells and postmortem brain tissue using TRI Reagent™ (Invitrogen™, Carlsbad, CA, USA, Cat. No. AM9738).

For cell samples, 1 mL of TRI Reagent™ was added to HEK293 control cells seeded 48 hours prior to RNA isolation in tissue culture-treated cell culture dishes (58 cm^2^; Greiner Bio-One, Cat. No. 664160). In oxidative stress experiments, HEK293 control cells were treated with 5 µM rotenone for 6 hours before RNA extraction.

For brain samples, approximately 100 mg of cortical tissue was dissected into uniformly sized pieces and homogenized using a mechanical tissue squisher in 1 mL of TRI Reagent™. Following homogenization, 200 µL of chloroform (Carl Roth, Karlsruhe, Germany, Cat. No. 3313.1) was added, mixed thoroughly, and centrifuged at 13,000 × g for 15 minutes at 4 °C. The resulting aqueous phase was transferred to a new reaction tube containing 1 µL of glycogen (Thermo Fisher Scientific™, Waltham, MA, USA, Cat. No. R0551) to aid RNA precipitation. RNA was precipitated by adding 1 mL of 100% 2-propanol (Carl Roth, Karlsruhe, Germany, Cat. No. 22CN.1), followed by centrifugation at 13,000 × g for 15 minutes at 4 °C. The RNA pellet was washed with 1 mL of 75% ethanol (Carl Roth, Karlsruhe, Germany, Cat. No. 9065.4) and centrifuged at 18,000 × g for 10 minutes at 4 °C. After discarding the supernatant, RNA pellets were air-dried and resuspended in RNase-free water (Invitrogen™, Carlsbad, CA, USA, Cat. No. AM9932). RNA concentration and purity were assessed using a NanoDrop spectrophotometer (Thermo Fisher Scientific™, Waltham, MA, USA).

*Western blot analysis*

Membranes were blocked with 5% bovine serum albumin (BSA; Carl Roth, Cat. No. T844.3) for 1 hour at room temperature or EveryBlot Blocking Buffer for 5 min. (Bio-Rad Laboratories, Hercules, CA, USA, Cat. No. 12010020) and incubated overnight at 4 °C with the following primary antibodies: anti-ANG (Abcam, Cambridge, UK, Cat. No. ab308074), anti-GAPDH (Abcam, Cambridge, UK, Cat. No. ab8245), anti-β-Actin (Sigma-Aldrich®, Cat. No. A1978), anti-NLRP1 (Abcam, Cambridge, UK, Cat. No. ab36852), and anti-NLRP3 (Abcam, Cambridge, UK, Cat. No. ab263899). Anti-NLRP1, and anti-NLRP3, were analyzed using Bio-Rad's Stain-Free™ technology. After washing with 0.5% Tris-buffered saline with Tween® 20 (TBST: 20 mM Tris, 150 mM NaCl, 0.5% Tween® 20; Carl Roth, Cat. No. 9127.1), membranes were incubated with HRP-conjugated secondary antibodies (anti-rabbit, Sigma-Aldrich®, Cat. No. A0545; or anti-mouse, Invitrogen, Cat. No. 31430) for 1 hour at room temperature. Signal detection was performed using Amersham ECL™ Prime Western Blotting Reagent (Cytiva, Cat. No. RNP2236) or Clarity Max Western ECL Substrate (Bio-Rad Laboratories, Hercules, CA, USA, Cat. No. 1705062) and imaged on a Fusion Pulse TS system (Vilber Lourmat, France) or a ChemiDoc Imaging System (Bio-Rad Laboratories, Hercules, CA, USA). Band sizes were analyzed as follows: ANG (14.1 kDa), NLRP1 (100 kDa), NLRP3 (130 kDa), GAPDH (36 kDa), and β-Actin (43 kDa). Densitometric analysis was performed using ImageLab software (Bio-Rad).

*Real-Time quantitative PCR (RT-qPCR)*

Total RNA was extracted using TRI Reagent™ (Invitrogen™, Carlsbad, CA, USA, Cat. No. AM9738) as described above. RNA purity was assessed via spectrophotometry, and only samples with A_260_/_280_ > 2.0 and A_260_/_230_ > 1.5 were used for downstream applications. cDNA was synthesized from high-quality RNA using the High-Capacity cDNA Reverse Transcription Kit (Applied Biosystems™, Foster City, CA, USA, Cat. No. 4368814) following the manufacturer’s protocol. qPCR was performed using TaqMan® Gene Expression Assays specific for ANG and β-Actin (Applied Biosystems™, Foster City, CA, USA), combined with TaqMan® Fast Advanced Master Mix (Applied Biosystems™, Foster City, CA, USA, Cat. No. 4444557) to prepare the reaction mixture. cDNA (2 µg in 20 µL) was diluted to 5 ng/µL prior to amplification. Reactions were prepared in triplicate for each target and endogenous control, and no-template controls were included to monitor for contamination. Reactions were assembled in 96-well optical plates and sealed with MicroAMP™ Optical Adhesive Film (Applied Biosystems™, Foster City, CA, USA, Cat. No. 4306311). Amplification and fluorescence detection were carried out using the QuantStudio™ 5 Real-Time PCR System (Thermo Fisher Scientific™, Waltham, MA, USA). Relative gene expression was calculated using the ∆∆CT method.

*Radioactive [^32^P]-labeled Northern blotting*

To detect specific tRNA-derived fragments, a customized probe targeting tiRNA^Gly-GCC^ (sequence available in the Supplementary Information Table S1) was used as described above. Total RNA was extracted from cultured cells and postmortem brain tissue using TRIzol™ reagent (Invitrogen™, Carlsbad, CA, USA, Cat. No. AM9738) and quantified using a NanoDrop spectrophotometer (Thermo Fisher Scientific, Waltham, MA, USA), following the manufacturer’s instructions. For each sample, 10 µg of total RNA were loaded onto a 15% denaturing polyacrylamide gel (8 M urea, 1× TBE) and electrophoresed at 300 V for 60 minutes. Following electrophoresis, RNA was transferred to a Hybond-N⁺ membrane (Cytiva, formerly GE Healthcare, Marlborough, MA, USA) using a semi-dry Trans-Blot Turbo Transfer System (Bio-Rad Laboratories, Hercules, CA; USA) at 15 V for 60 minutes. To immobilize RNA, membranes were UV-crosslinked at 120 mJ/cm² using a Stratalinker 1800 (Stratagene UV Stratalinker 1800, CA, USA) and dried at 80 °C for 30 minutes before hybridization. Membranes were pre-hybridized for 1 hour at 42 °C in hybridization buffer (0.5 M sodium phosphate, 1 mM EDTA pH 8, 7% w/v SDS) supplemented with 0.2 mg/mL salmon sperm DNA (Hybrid Mini 38, H. Saur). A tRNA fragment-specific probe was 5′ end-labeled with [γ-^32^P] ATP using T4 polynucleotide kinase (Thermo Fisher Scientific, Waltham, MA, USA) similarly to [5]. In brief, the labeling reaction contained 1.6 pmol oligonucleotide, 1× reaction buffer A, 1.6 pmol [γ-^32^P] ATP, and 0.5 U/µL T4 PNK, and was incubated for 30 minutes at 37 °C. The reaction was terminated by adding EDTA (pH 8.0), followed by heat inactivation at 75°C for 10 minutes according to manufacturer’s instructions. Labeled probes were hybridized to membranes overnight, followed by three high-stringency washes (1M sodium phosphate, 1 mM EDTA pH 8, 1% w/v SDS) to remove non-specific binding. Signals were detected using a Typhoon FLA9500 phosphorimager (Cytiva, formerly GE Healthcare, Marlborough, MA, USA) with an exposure time of 6h for each membrane, and band intensities were quantified with ImageLab software (Bio-Rad Laboratories, Hercules, CA, USA) to assess expression levels of the targeted tRNA fragment.

*Caspase-Glo® 3/7 Assay*

To investigate the protective effect of ANG, HEK293 control (HEK293ut) and APPwt cells were seeded at 4 × 10⁴ cells per well in white 96-well plates 24 hours prior to treatment. Untreated HEK293ut and APPwt cells served as controls for basal caspase-3/7 activity.

Cells were treated with recombinant human ANG (rANG; 0.5 µg/mL; Sigma-Aldrich®, Cat. No. A6955) for 2 hours. To assess oxidative stress-induced apoptosis, 5 µM rotenone was administered for either 6 or 24 hours, either 2 hours before or after rANG treatment. For experiments assessing the role of 5′-tiRNA^Gly-GCC^, cells were treated for 24 hours with either a synthetic 5′-tiRNA^Gly-GCC^ mimic or a specific inhibitor (sequences provided in the Supplementary Information). In these experiments, rANG was added 2 hours before application of the respective stressor. All treatments were conducted in standard culture medium adjusted to pH 7.4. Caspase-3/7 activity was measured using the Caspase-Glo® 3/7 Assay Kit (Promega, Madison, WI, USA, Cat. No. G8091), following the manufacturer’s protocol. This bioluminescent assay uses a DEVD-aminoluciferin substrate cleaved by active caspase-3/7, releasing aminoluciferin, which is then metabolized by luciferase to generate a luminescent signal proportional to caspase activity. Luminescence was recorded using a multiwell plate reader (FlexStation, Molecular Devices, UK).

*Transcriptomic analysis*

Gene expression data were obtained from the ROSMAP study via Synapse.org (<https://www.synapse.org/Synapse:syn23446022>). For initial analysis, FPKM values of selected genes (see Supplementary Table S3 fore detailed information) were log_2_ fold-change (log_2_FC) transformed relative to non-demented controls (NDC), and visualized as heatmaps using GraphPad Prism (v10.4.2). To explore molecular heterogeneity, Uniform Manifold Approximation and Projection (UMAP) was applied to protein expression data from the ROSMAP cohort. Prior to analysis, formatting inconsistencies were resolved (e.g., decimal separator standardization, replacement of non-numeric entries). Gene values were z-score normalized before UMAP dimensionality reduction using the umap-learn Python library (v0.5.3) with 15 nearest neighbors and a minimum distance of 0.1. Visualization was performed in 2D using matplotlib (v3.7.1) and seaborn (v0.12.2). Key Python libraries included pandas (v2.0.3) and NumPy (v1.24.3). Clusters in the 2D UMAP embedding were identified using Hierarchical Density-Based Spatial Clustering of Applications with Noise (HDBSCAN) (v0.8+), with a minimum cluster size of 10, minimum samples of 5, ε = 0.5 and Euclidean distance as the metric. HDBSCAN, an unsupervised density-based clustering algorithm that does not require predefinition of cluster number, was applied to molecular expression features from the transcriptomic dataset to identify subtypes based solely on the intrinsic structure of the data. Thus, subtype classification was entirely data-driven, without predefined expression thresholds or forced cluster numbers, and was performed independently of all downstream clinical outcome variables, including diagnosis, cognitive decline trajectories, and survival endpoints. A cluster label of -1 was assigned to noise points. Cluster distributions were visualized by coloring UMAP embeddings according to HDBSCAN labels. Following unsupervised clustering, ANG expression levels were compared across the resulting subtypes to assess whether ANG abundance differed significantly between clusters. To assess robustness, the clustering approach was applied across independent datasets, including ROSMAP (cohort 1), the NBB postmortem brain tissue mRNA-seq dataset (cohort 2), and the ADTBI cohort (cohort 3), using the same analytical pipeline. As Cohort 2 (NBB) consisted of only six individual patients, the minimum cluster size parameter was adjusted accordingly to allow inclusion of all samples in the clustering analysis. Following cluster assignment in each cohort, gene expression profiles were examined within each group. Special focus was given to inflammatory markers and microglial proteins to characterize immune-related heterogeneity. Across all cohorts, the identified subtypes showed consistent subtype-specific transcriptional signatures. Importantly, ANG expression levels within each assigned cluster were comparable across cohorts and mirrored the patterns observed in the ROSMAP cohort (Supplementary Fig. S8), further supporting the robustness and biological relevance of the identified subtypes. Sensitivity analyses indicated that lowering cluster_selection_epsilon or min_cluster_size increased cluster granularity, resulting in additional microclusters as expected. Notably, these additional clusters represented subdivisions within the originally defined states rather than a change in the overarching subtype architecture, as samples continued to segregate into groups with consistently low versus high ANG expression. This preservation of the principal biological partitioning supports the three-cluster solution as the most parsimonious higher-level representation of the data (Supplementary Fig. S8). Overall survival times and Mini-Mental State Examination (MMSE) scores were used to evaluate cognitive trajectories. Individuals for whom no MMSE was documented at study inclusion, at the time of AD diagnosis, or prior to death were excluded from our analysis, as no comparison between the time of AD diagnosis and the last visit before death was possible for these patients.

*GO Pathway analysis*

Gene Ontology (GO) over-representation analysis was performed with g:Profiler’s g:GOSt service (using Python client v1.0.0 and API build 2024-05-17). ENSEMBL gene identifiers (GRCh38, release 113) derived from each protein cluster were used as input. Lists were queried against the *Homo sapiens* reference background supplied by g:Profiler. We interrogated the Biological Process, Molecular Function, and Cellular Component branches, as well as KEGG pathways. The search parameters were: significance threshold 0.05, Benjamini–Hochberg false-discovery-rate correction, inclusion of both experimental and electronic annotations, and enrichment calculated relative to the whole-genome background. Result tables containing term ID, description, intersecting genes, term size, raw p value and FDR-adjusted p value were downloaded and collated with pandas 2.2.2 into Excel workbooks (separate sheet per cluster) and a consolidated CSV file. For visualization, -log_10_ FDR was computed. Values derived from gene sets with downregulated pathway scores were then multiplied by -1 to generate mirrored bar and dot plots. Graphs were produced with GraphPad Prism (GraphPad Software, La Jolla, CA, USA).

*Survival rates and hazard ratios*

Kaplan-Meier survival curves with Greenwood 95 % confidence bands were generated for each subtype using lifelines 0.25.11 in Python 3.11. Group differences were tested with two-sided log-rank tests. A Cox proportional-hazards model, with subtype as a categorical predictor (reference=Subtype 1), provided hazard ratios (HR) and 95 % Wald confidence intervals; p < 0.05 denoted significance. Cox proportional hazards assumptions were formally assessed using Schoenfeld residual–based tests. No evidence of violation of the proportional hazards assumption was observed, either globally (*X*^2^ = 2.11, df = 2, *p* = 0.349) or for the individual subtype terms (Subtype 1 vs Subtype 0, *p* = 0.887; Subtype 2 vs Subtype 0, *p* = 0.149). In addition, given the modest sample size, survival differences were further evaluated using complementary non-parametric approaches, including the log-rank (Mantel-Cox) test and the log-rank test for trend.

*Statistical analysis*

All data were analyzed using standard statistical procedures in GraphPad Prism v10.4.2 (GraphPad Software, La Jolla, CA, USA). More information is provided in Supplementary Information. Unpaired Student’s t-tests were used for comparisons between two groups, while one-way ANOVA, followed by appropriate post hoc test (Turkey’s multiple comparisons test), was applied for comparisons involving more than two groups. Unless otherwise specified (e.g., in heatmaps or transcriptomic analyses), results are presented as mean ± SEM. A minimum of three biological replicates was included per experiment. Before hypothesis testing, datasets were assessed for normality to ensure the appropriate statistical method was applied.

**Supplementary Table S1.** Sequences for hybridization probe and synthetic tRF modulators

| **Description** | **tRNA/tRF** | **Anticodon** | **Sequence** |
| --- | --- | --- | --- |
| Northern Blot hybridization probe | Gly | GCC | 5′-AGGCGAGAATTCTACCACTGAACCACCAATGC-3′ |
| tRF mimic | Gly | GCC | 5'-GCA UUG GUG GUU CAG UGG UAG AAU UCU CGC C-3 |
| tRF inhibitor | Gly | GCC | 5'-CGU AAC CAC CAA GUC ACC AUC UUA AGA GCG G-3' |

**Supplementary Table S2.** Patient details of the NBB postmortem brain tissue.

| Individuum | Diagnosis | Sex | Age [years] | Braak stage |
| --- | --- | --- | --- | --- |
| 1 | AD | female | 88 | 5 |
| 2 | AD | female | 86 | 5 |
| 3 | AD | female | 81 | 6 |
| 4 | AD | female | 88 | 4 |
| 5 | AD | female | 72 | 5 |
| 6 | AD | female | 98 | 5 |
| 7 | AD | female | 87 | 4 |
| 8 | AD | female | 76 | 4 |
| 9 | AD | male | 80 | 4 |
| 10 | AD | male | 78 | 5 |
| 11 | AD | male | 86 | 4 |
| 12 | AD | male | 97 | 5 |
| 13 | AD | male | 74 | 5 |
| 14 | AD | male | 80 | 4 |
| 15 | Non-demented control | female | 83 | 2 |
| 16 | Non-demented control | female | 90 | 3 |
| 17 | Non-demented control | female | 76 | 2 |
| 18 | Non-demented control | female | 71 | 2 |
| 19 | Non-demented control | female | 85 | 3 |
| 20 | Non-demented control | female | 84 | 3 |
| 21 | Non-demented control | male | 92 | 4 |
| 22 | Non-demented control | male | 102 | 3 |
| 23 | Non-demented control | male | 93 | 0 |
| 24 | Non-demented control | male | 72 | 2 |
| 25 | Non-demented control | male | 86 | 3 |
| 26 | Non-demented control | male | 87 | 3 |

**Supplementary Table S3.** Details of searched proteins for subtyping experiments.

**Supplementary Table S4.** AD subtype differences in survival rates across subtypes 1-3

**Supplementary Table S5.** AD subtype comparison on MMSE scores

**Supplementary Table S6.** Subtype comparison on ApoE genetic risk profiles

**Supplementary Table S7.** Subtype comparison education, ethnicity and origin

**Supplementary Methods for experimental procedures described in the Supplementary Figures manuscript**

*Animals (Supplements)*

Transgenic 5xFAD mice were obtained from and housed at the Translational Animal Research Center (TARC) at the University Medical Center Mainz, Germany. Animals used in this study were sex-mixed (male and female) and aged either 3- or 12 months. Housing and care conditions followed protocols described by Brandscheid et al. [6, 7]. Mice were euthanized by cervical dislocation or decapitation under isoflurane anesthesia (Isothesia, Piramal Critical Care B.V.). All procedures were conducted in accordance with the European Communities Council Directive (2010/63/EU), the ARRIVE guidelines and the local animal ethics committee guidelines (Landesuntersuchungsamt Rheinland-Pfalz, LUA).

*Preparation of protein extracts*

The hippocampus and cortex were freshly dissected and rinsed with PBS to remove residual blood. Depending on tissue size, 200–1000 µL of RIPA buffer were added for lysis. Tissue homogenates were incubated at room temperature for 45 minutes and subsequently centrifuged at 10,000 × g for 10 minutes at 4 °C. The resulting supernatant, containing soluble proteins, was collected. Protein concentrations were determined using the Bradford assay (Bio-Rad Protein Assay Dye Reagent Concentrate, Bio-Rad Laboratories, Cat. No. 5000006) according to the manufacturer’s instructions.

*Validation of anti-ANG antibody*

For validation of the antibody mouse liver tissue and HEK293 control cell protein lysates were used and prepared as described previously. To validate the specificity of the polyclonal anti-ANG antibody, a blocking peptide competition assay was performed. The anti-ANG antibody (Abcam, Cambridge, UK, Cat. No. ab308074) was pre-incubated with a 5-fold molar excess of the ANG blocking peptide (amino acid sequence corresponding to the immunogen) in PBS with 1% BSA for 1 hour at room temperature. The peptide-antibody mixture was then applied to Western blot membranes or immunofluorescence samples in parallel with control samples incubated with the antibody alone. A marked reduction or absence of ANG signal in the presence of the blocking peptide confirmed the antibody's specificity. For all experiments, equal exposure times and identical imaging settings were used to ensure valid comparisons.

*Aβ_1-40_ ELISA*

Aß_1-40_ levels were quantified using the Amyloid Beta 40 Human ELISA Kit (Invitrogen, KHB3482). Supernatants were collected from HEK control, APPwt, and DAPT-treated cells after 24 hours of treatment. The ELISA was performed following the manufacturer's protocol

*Statistical analysis*

All data were analyzed using standard statistical procedures in GraphPad Prism v10.4.2 (GraphPad Software, La Jolla, CA, USA). Unpaired two-tailed t-tests were used for comparisons between two groups, while one-way ANOVA was applied for comparisons involving more than two groups. Normality of data distributions was evaluated prior to statistical testing. Unless otherwise specified (e.g., in heatmaps or transcriptomic analyses), results are presented as mean ± SEM. A minimum of three biological replicates was included per experiment. Prior to hypothesis testing, datasets were assessed for normality to ensure the appropriate statistical method was applied.

**Uncropped Images**

**Uncropped Figure 1.** Full Western blot membrane showing male postmortem brain tissue samples from non-demented control (NDC) and Alzheimer’s disease (AD) cases obtained from the Netherlands Brain Bank (NBB). This blot corresponds to the cropped version presented in Figure 1d. All samples were processed and imaged under identical conditions to allow direct comparison. Antibody dilutions are provided in the Materials and Methods section. Exposure time ANG: ~ 2 min; β-Actin: ~30 sec.

**Uncropped Figure 2.** Full Western blot membrane showing female postmortem brain tissue samples from non-demented control (NDC) and Alzheimer’s disease (AD) cases obtained from the Netherlands Brain Bank (NBB). This blot corresponds to the cropped version presented in Figure 1e. Regions used in the main figure are highlighted in purple. All samples were processed and imaged under identical conditions to allow direct comparison. Antibody dilutions are provided in the Materials and Methods section. Exposure time ANG: ~ 2 min; β-Actin: ~30 sec.

**Uncropped Figure 3.** Full [^32^P]-labeled Northern blot of male NBB postmortem brain samples used in Alzheimer’s disease (AD) experiments. The upper band corresponds to full-length tRNA^Gly-GCC^, and the lower band represents the 5′ tRNA-derived fragment (5′-tiRNA^Gly-GCC^). This is the uncropped version of the blot shown in Figure 1h. Exposure and membrane processing were consistent across samples to ensure comparability.

**Uncropped Figure 4.** Full [^32^P]-labeled Northern blot of female NBB postmortem brain samples used in aging and Alzheimer’s disease (AD) experiments. The upper band corresponds to full-length tRNA^Gly-GCC^, while the lower band represents the 5′ tRNA-derived fragment (5′-tiRNA^Gly-GCC^). Blot is shown in its uncropped form, corresponding to the version presented in Figure 1i. All samples were processed and exposed under identical conditions to ensure comparability.

**Uncropped Figure 5.** Full Western blot membrane of HEK293 control cells treated with or without rotenone. The membrane was cut directly above the molecular weight marker corresponding to ANG prior to antibody incubation; the section containing β-Actin was also separated for individual probing with anti-β-actin antibody. This blot corresponds to the cropped version shown in Figure 2a. Regions used in the main figure are highlighted in purple. All samples were processed and imaged under identical conditions to allow direct comparison. Antibody dilutions are provided in the Materials and Methods section. Exposure time ANG: ~ 2 min; β-Actin: ~30 sec.

**Uncropped Figure 6.** Full Western blot membrane of HEK293 control cells treated with or without rotenone and/or vitamin C. This blot corresponds to the cropped version presented in Figure 2c. Cropped regions used in the main figure are highlighted in purple. All samples were processed and imaged under identical conditions to allow direct comparison. Antibody dilutions are provided in the Materials and Methods section. Exposure time ANG: ~ 2 min; β-Actin: ~30 sec.

**Uncropped Figure 7.** Full [^32^P]-labeled Northern blot of HEK293 control cells treated with or without rotenone. The upper band corresponds to full-length tRNA^Gly-GCC^, while the lower band represents the 5′ tRNA-derived fragment (5′-tiRNA^Gly-GCC^). This blot corresponds to the cropped version shown in Figure 2h. Regions included in the main figure are highlighted in purple. All samples were run, transferred, and exposed under identical conditions to ensure accurate comparison.

**Uncropped Figure 8.** Full Western blot membrane of HEK293 control and APPwt cells, revealing ANG basal protein expression. This blot corresponds to the cropped version shown in Figure 2i. Cropped regions used in the main figure are highlighted in purple. All samples were processed and imaged under identical conditions to allow direct comparison. Antibody dilutions are provided in the Materials and Methods section. Exposure time ANG: ~ 2 min; β-Actin: ~30 sec.

**Uncropped Figure 9.** Full Western blot membrane of HEK293 control cells treated with or without oligomeric Aß_1-42_. This blot corresponds to the cropped version shown in Figure 2j. Cropped regions used in the main figure are highlighted in purple. All samples were processed and imaged under identical conditions to allow direct comparison. Antibody dilutions are provided in the Materials and Methods section. Exposure time ANG: ~ 2 min; β-Actin: ~30 sec.

**Uncropped Figure 10.** Full Western blot membrane of HEK293 control and APPwt cells treated with or without DAPT. This blot corresponds to the cropped version shown in Figure 2k. Cropped regions included in the main figure are highlighted in purple. For this blot, β-Actin was probed first. The membrane was then stripped using stripping buffer for 30 minutes at 55 °C to remove the β-Actin antibody signal before re-probing for ANG. Since a faint β-Actin band was still visible after stripping, the membrane was cut above the 25 kDa molecular weight marker to remove the residual β-Actin signal and avoid interference with ANG detection. To verify proper marker alignment, both blot images (ANG and β-Actin) were overlaid, demonstrating consistent marker positions across probes. All samples were processed and imaged under identical conditions to allow direct comparison. Antibody dilutions are provided in the Materials and Methods section. Exposure time ANG: ~ 2 min; β-Actin: ~30 sec.

**Uncropped Figure 11.** Full Western blot membrane of APPwt cells treated with or without vitamin C. This blot corresponds to the cropped version shown in Figure 2l. Regions included in the main figure are highlighted in purple. All samples were processed and imaged under identical conditions to allow direct comparison. Antibody dilutions are provided in the Materials and Methods section. Exposure time ANG: ~ 2 min; β-Actin: ~30 sec.

**Uncropped Figure 12.** Full [^32^P]-labeled Northern blot of HEK293 control and APPwt cells. The upper band corresponds to full-length tRNA^Gly-GCC^, and the lower band represents the 5′ tRNA-derived fragment (5′-tiRNA^Gly-GCC^). This blot corresponds to the cropped version shown in Figure 2o. Cropped regions included in the main figure are highlighted in purple. All samples were run, transferred, and exposed under identical conditions to ensure accurate comparison.

**Uncropped Figure 13.** Full Western blot membrane of human SH-SY5Y cells treated with or without oligomeric Aβ_1-42_. This blot corresponds to the cropped version shown in Figure 3a. Protein normalization was performed using total protein levels visualized with Bio-Rad Stain-Free™ technology. Cropped regions included in the main figure are highlighted in purple. All samples were processed and imaged under identical conditions to allow direct comparison. Antibody dilutions are provided in the Materials and Methods section. Exposure time ANG: ~ 2 min; β-Actin: ~30 sec.

**Uncropped Figure 14.** Full Western blot membrane of male NBB non-demented control (NDC) and Alzheimer’s disease (AD) brain tissue samples. This blot corresponds to the cropped version shown in Figure 3b. Protein normalization was performed using total protein levels visualized with Bio-Rad Stain-Free™ technology. Cropped regions included in the main figure are highlighted in purple. All samples were processed and imaged under identical conditions to allow direct comparison. Antibody dilutions are provided in the Materials and Methods section. Exposure time NLRP1: ~ 30 sec..

**Uncropped Figure 15.** Full Western blot membrane showing NLRP3 expression in male NBB non-demented control (NDC) and Alzheimer’s disease (AD) brain tissue samples. This blot corresponds to the cropped version presented in Figure 3c. Protein normalization was performed using total protein levels visualized with Bio-Rad Stain-Free™ technology. Cropped regions included in the main figure are highlighted in purple. All samples were processed and imaged under identical conditions to allow direct comparison. Antibody dilutions are provided in the Materials and Methods section. Exposure time NLRP3: ~ 50 sec..

**Uncropped Figure 16.** Full Western blot membrane showing NLRP1 expression in female NBB non-demented control (NDC) and Alzheimer’s disease (AD) brain tissue samples. This blot corresponds to the cropped version presented in Figure 3d. Protein normalization was performed using total protein levels visualized with Bio-Rad Stain-Free™ technology. Cropped regions shown in the main figure are highlighted in purple. All samples were processed and imaged under identical conditions to allow direct comparison. Antibody dilutions are provided in the Materials and Methods section. Exposure time NLRP1: ~ 30 sec..

**Uncropped Figure 17.** Full Western blot membrane showing NLRP3 expression in female NBB non-demented control (NDC) and Alzheimer’s disease (AD) brain tissue samples. This blot corresponds to the cropped version presented in Figure 3e. Protein normalization was performed using total protein levels visualized with Bio-Rad Stain-Free™ technology. Cropped regions shown in the main figure are highlighted in purple. All samples were processed and imaged under identical conditions to allow direct comparison. Antibody dilutions are provided in the Materials and Methods section. Exposure time NLRP3: ~ 50 sec..

**Supplementary Bibliography**

1.	Miller JA, Guillozet-Bongaarts A, Gibbons LE, Postupna N, Renz A, Beller AE, et al. Neuropathological and transcriptomic characteristics of the aged brain. Elife. 2017;6.

2.	Leuner K, Schütt T, Kurz C, Eckert SH, Schiller C, Occhipinti A, et al. Mitochondrion-derived reactive oxygen species lead to enhanced amyloid beta formation. Antioxid Redox Signal. 2012. 2012. https://doi.org/10.1089/ars.2011.4173.

3.	Eckert A, Steiner B, Marques C, Leutz S, Romig H, Haass C, et al. Elevated vulnerability to oxidative stress-induced cell death and activation of caspase-3 by the Swedish amyloid precursor protein mutation. J Neurosci Res. 2001;64:183–192.

4.	Stine WB, Jungbauer L, Yu C, Ladu MJ. Preparing synthetic Aβ in different aggregation states. Methods in Molecular Biology. 2011;670:13–32.

5.	Müller M, Tittel LS, Petfalski E, Iyer KV, Kraft A-A, Pastore S, et al. Yeast elongation factor homolog New1 protects a subset of mRNAs from degradation by no-go decay. Nucleic Acids Res. 2026;54:gkag047.

6.	Oakley H, Cole SL, Logan S, Maus E, Shao P, Craft J, et al. Intraneuronal β-Amyloid Aggregates, Neurodegeneration, and Neuron Loss in Transgenic Mice with Five Familial Alzheimers Disease Mutations: Potential Factors in Amyloid Plaque Formation. The Journal of Neuroscience. 2006;26:10129 LP – 10140.

7.	Brandscheid C, Schuck F, Reinhardt S, Schäfer K-H, Pietrzik CU, Grimm M, et al. Altered Gut Microbiome Composition and Tryptic Activity of the 5xFAD Alzheimer’s Mouse Model. Journal of Alzheimer’s Disease. 2017;56:775–788.
